# Apollon: a deoxyribozyme that generates a yellow product

**DOI:** 10.1093/nar/gkae490

**Published:** 2024-06-13

**Authors:** Martin Volek, Jaroslav Kurfürst, Milan Kožíšek, Pavel Srb, Václav Veverka, Edward A Curtis

**Affiliations:** Institute of Organic Chemistry and Biochemistry of the Czech Academy of Sciences, Prague 166 10, Czech Republic; Department of Genetics and Microbiology, Faculty of Science, Charles University in Prague, Prague 128 44, Czech Republic; Institute of Organic Chemistry and Biochemistry of the Czech Academy of Sciences, Prague 166 10, Czech Republic; Department of Informatics and Chemistry, University of Chemistry and Technology, Prague 166 28, Czech Republic; Institute of Organic Chemistry and Biochemistry of the Czech Academy of Sciences, Prague 166 10, Czech Republic; Institute of Organic Chemistry and Biochemistry of the Czech Academy of Sciences, Prague 166 10, Czech Republic; Institute of Organic Chemistry and Biochemistry of the Czech Academy of Sciences, Prague 166 10, Czech Republic; Department of Cell Biology, Faculty of Science, Charles University in Prague, Prague 128 44, Czech Republic; Institute of Organic Chemistry and Biochemistry of the Czech Academy of Sciences, Prague 166 10, Czech Republic

## Abstract

Colorimetric assays in which the color of a solution changes in the presence of an input provide a simple and inexpensive way to monitor experimental readouts. In this study we used *in vitro* selection to identify a self-phosphorylating kinase deoxyribozyme that produces a colorimetric signal by converting the colorless substrate pNPP into the yellow product pNP. The minimized catalytic core, sequence requirements, secondary structure, and buffer requirements of this deoxyribozyme, which we named Apollon, were characterized using a variety of techniques including reselection experiments, high-throughput sequencing, comparative analysis, biochemical activity assays, and NMR. A bimolecular version of Apollon catalyzed multiple turnover phosphorylation and amplified the colorimetric signal. Engineered versions of Apollon could detect oligonucleotides with specific sequences as well as several different types of nucleases in homogenous assays that can be performed in a single tube without the need for washes or purifications. We anticipate that Apollon will be particularly useful to reduce costs in high-throughput screens and for applications in which specialized equipment is not available.

## Graphical Abstract

**Graphical Abstract:**
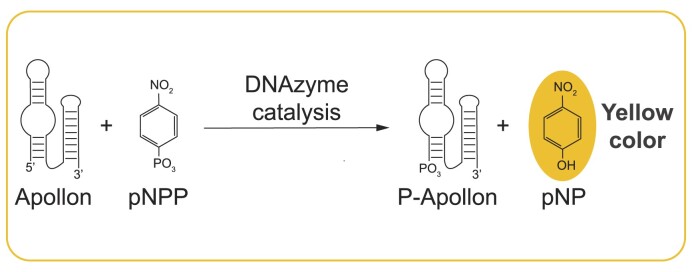

## Introduction

Nucleic acids play important roles in the storage and transfer of genetic information, but are also capable of many other functions. These include the ability to catalyze chemical reactions. Functional nucleic acid motifs are typically identified using methods of artificial evolution such as SELEX and *in vitro* selection (1–3). The starting point in a standard selection experiment is a large library of DNA or RNA molecules containing randomized regions flanked by primer binding sites. After enriching the population for rare molecules with a desired phenotype, surviving library members are amplified by PCR or RT-PCR. By performing multiple rounds of selection, it is possible to purify rare sequences with a desired property from libraries of 10^14^ (or more) sequences. Such selection experiments have been used to isolate many different types of aptamers (DNA and RNA molecules that bind ligands), ribozymes (catalytic RNA molecules), and deoxyribozymes (catalytic DNA molecules) (4–9). Functional DNA and RNA molecules identified in such experiments can be rapidly synthesized (often in less than a day) using well-developed chemical and enzymatic methods. They are easier and less expensive to manufacture than proteins, and can be more readily optimized and modified in reselection experiments. These typically involve making a second library by random mutagenesis of a single isolate of a functional motif, performing a second selection experiment, and characterizing the active variants by high-throughput sequencing and comparative analysis. An additional advantage of functional DNA is that it is extremely stable, especially compared to RNA.

Our group is interested in developing deoxyribozymes that can be used for diagnostic applications, and in recent studies we identified catalytic DNA molecules motifs that generate chemiluminescent and fluorescent products. Our approach uses substrates that generate signals when they are dephosphorylated in combination with kinase deoxyribozymes that dephosphorylate these substrates. One deoxyribozyme identified using this method, called Supernova, generates blue light by transferring the phosphate group from the substrate CDP-Star to its own 5′ hydroxyl group (10,11). Another, called Aurora, generates purple fluorescence by transferring the phosphate group from the substrate 4-MUP to itself (12). These deoxyribozymes can be engineered to only generate signals in the presence of specific input molecules. For example, an engineered version of Supernova is only active in the presence of oligonucleotides with specific sequences and can be programmed to detect a wide variety of target oligonucleotides (10). Another type of sensor is activated by cleavage of a single RNA linkage at its 5′ end, and can be used to detect ribonucleases with a detection limit of approximately 100 pM (12). Because such sensors can be used in solution without the need for washes or purifications, they show great promise for applications such as high-throughput screens (13) and point-of-care assays (14).

Deoxyribozymes that generate chemiluminescent or fluorescent signals such as Supernova and Aurora are good choices for certain assays because of their high sensitivity, but expensive instrumentation is needed to analyze the results. In comparison, assays that monitor changes in the color of a sample are less sensitive, but can be analyzed without expensive equipment and sometimes even by eye. The SARS-CoV-2 pandemic highlighted the utility of such colorimetric assays in diagnostics: although RT-PCR is a much more sensitive method, tests were frequently performed using faster and cheaper antigen assays that utilize a colorimetric readout (15). More generally, colorimetric assays are especially useful in cases in which instruments like fluorescent plate readers are not available or cannot be easily used.

Inspired by the advantages of such assays, in this study we set out to identify catalytic DNA motifs that catalyze a colorimetric reaction. To identify such deoxyribozymes, a selection was performed for DNA molecules that transfer a phosphate group (16) from the substrate 4-nitrophenyl phosphate (pNPP) to their own 5′ hydroxyl group. Dephosphorylation of pNPP generates a yellow product that can be detected using a spectrophotometer or by eye (17). The sequence requirements of one of these catalytic motifs, which we named Apollon, were further characterized using a combination of reselection, high-throughput sequencing, and comparative analysis, and its buffer requirements were investigated using both biochemical assays and proton NMR. Like Supernova (10,11) and Aurora (12), the minimized core of Apollon is <50 nucleotides long, it requires multiple zinc ions for activity, and it catalyzes self-phosphorylation with a catalytic efficiency (*k*_cat_/K_M_) of about 200 M^−1^ min^−1^ and a rate (*k*_obs_) of 0.3 min^−1^ at 1.5 mM pNPP. By separating the deoxyribozyme into a substrate strand (which contains the reaction site) and an enzyme strand (which contains the rest of the deoxyribozyme), it was possible to develop a variant that catalyzed multiple turnover phosphorylation and amplified the colorimetric signal. Homogenous assays were also developed that can be used to detect oligonucleotides with specific sequences, ribonucleases that generate 2′,3′-cyclic phosphate and 5′ hydroxyl termini, and nucleases such as DNase I. Because the product of this reaction can be visualized by eye or using widely available devices such as smartphones, we anticipate that Apollon will be particularly useful for applications in which expensive instruments such as fluorescent plate readers are not available.

## Materials and methods

### Oligonucleotides

Oligonucleotides were chemically synthesized by GENERI BIOTECH s.r.o., Eurofins Genomics or IDT. Apollon 1 and 2 were purified by HPLC, and other oligonucleotides were typically purified by 6% denaturing PAGE. See Supplementary Table S1 for the sequences of oligonucleotides and deoxyribozymes used in this study.

### Pool design

The library used in our initial selection (see Pool1 in Supplementary Table S1) was generated by randomly mutagenizing the 85-nucleotide long H1 variant of Supernova (a chemiluminescent deoxyribozyme previously discovered in our group (10)) at a rate of 21% per position. A 20-nucleotide-long primer-binding site was also attached to its 3′ end. The library used for the reselection (see Pool2 in Supplementary Table S1) was based on the sequence of Hit 1 from initial selection. Hit 1 produced color with the highest signal-to-noise ratio of any of the tested deoxyribozymes from the initial selection, and it also had the highest read number in the high-throughput sequencing data. The 85-nucleotide long sequence of Hit 1 was randomly mutagenized at a rate of 21% per position. A new 20-nucleotide-long primer-binding site was also attached to its 3′ end to reduce the likelihood that contaminants from the initial selection could be amplified in the reselection.

### Initial selection

Pool1 and blocking oligonucleotide (see REV1 in Supplementary Table S1) were mixed in Milli-Q water, heated at 65°C for 2 min, and cooled at room temperature for 5 min. 5 × selection buffer and the disodium salt of 4-nitrophenyl phosphate (pNPP) were then added. Final concentrations were 1 μM Pool1, 1.5 μM REV1, 1 × selection buffer (200 mM KCl, 1 mM ZnCl_2_, 1 μM Ce(NO_3_)_4_, 0.1 μM PbCl_2_ and 50 mM HEPES pH 7.4) and 1 mM pNPP. After incubation for 2.4 h, DNA was purified by ethanol precipitation. A 20-nucleotide-long oligonucleotide (see FWD1 in Supplementary Table S1) was then ligated to Pool1 library members containing a 5′ phosphate. To ensure effective ligation, the reaction was performed with a 35-nucleotide-long splint oligonucleotide (see Splint1 in Supplementary Table S1) which was partially complementary to both FWD1 and the 5′ end of Pool1. FWD1, Pool1, and Splint1 were mixed with Milli-Q water, heated at 65°C for 2 min, and cooled at room temperature for 5 min. 10 × T4 DNA ligase buffer and T4 DNA ligase were added. Final concentrations were 2.5 μM Pool1, 2.5 μM FWD1, 2.5 μM Splint1, 1 × T4 DNA ligase buffer, and 0.5 Weiss units of T4 DNA ligase per 1.0 μg of Pool1. The ligation mixture was then incubated for 5 min at 37°C. Ligated DNA molecules were then separated from un-ligated ones by 6% denaturing PAGE. DNA molecules that co-migrated with a 125-nucleotide-long marker were cut from the gel, eluted into 0.3 M NaCl solution, mixed with 5 μg of yeast tRNA, which served as a carrier, and concentrated by ethanol precipitation. DNA was then amplified by PCR using Q5 HotStart DNA Polymerase and the FWD1r and REV1p primers (Supplementary Table S1). Final concentrations were 500-fold diluted DNA molecules (corresponding to reacted library members from Pool1 which had undergone ligation), 0.5 μM FWD1r, 0.5 REV1p, 1 × Q5 reaction buffer, 1 × Q5 high GC enhancer, 0.2 mM dNTPs, and 0.02 U Q5 HotStart DNA polymerase per 1.0 μl of the PCR reaction mixture. Double-stranded PCR products were purified using a Macherey-Nagel PCR Clean-up kit with NTI binding buffer that contained 30–45% guanidium thiocyanate. Because the strand complementary to Pool1 was amplified using REV1p, which contains a 5′ phosphate, it was possible to regenerate the single-stranded DNA Pool1 by digesting this strand with λ-exonuclease. This was achieved by incubating each 5 μg of double-stranded PCR product with Milli-Q water, 1 × Lambda exonuclease reaction buffer, and 1 μl (5 U) of Lambda exonuclease in a volume of 50 μl. This reaction was incubated at 37°C for 60 min. The resulting 125-nucleotide-long single-stranded DNA molecules were purified using a Macherey-Nagel PCR Clean-up kit with NTC binding buffer that contained 42–60% potassium thiocyanate. The last step was to regenerate the 5′ end of the Pool1 so it could undergo another round of selection. This was facilitated by the RNA linkage at the 3′ end of the FWD1r primer used in the PCR, which can be cleaved by base to remove the FWD1r primer and generate fragments with 2′,3′-cyclic phosphate and 5′-hydroxyl termini (16). To cleave this linkage, the 125-nucleotide long single-stranded DNA molecules were first heated in Milli-Q water at 65°C for 2 min and cooled at room temperature for 5 min, and adding 10 × hydrolysis buffer (1 × hydrolysis buffer contained 20 mM Trizma base, 400 mM KOH and 4 mM EDTA). The reaction mixture was incubated at 90°C for 10 min. The resulting 105-nucleotide-long DNA molecules (each containing a 5′ hydroxyl group) were isolated by 6% denaturing PAGE, eluted into 0.3 M NaCl, and concentrated by ethanol precipitation. Five rounds of this protocol were performed. The evolved library was then amplified by PCR, purified using a Macherey-Nagel PCR Clean-up kit, and sequenced by Eurofins Genomics using a standard amplicon paired-end sequencing run (e.g both strands of the DNA were sequenced).

### Reselection

Reselection conditions were the same as those used in the initial selection except for the following differences. First, Pool2 was used instead of Pool1. Second, Pool2 was incubated with pNPP for 14.4 min rather than 2.4 hours. Third, a different reverse primer (REV2/REV2p) was used (REV2p is a version of REV2 with a 5′ phosphate). Fourth, a different blocking oligonucleotide was used (Supplementary Table S1). The Pool2 was sequenced after seven rounds of selection by Eurofins Genomics using an amplicon paired-end sequencing run.

### Analysis of color production

To measure formation of the yellow product pNP, oligonucleotides corresponding to individual sequences from evolved libraries were resuspended in Milli-Q water, heated at 65°C for 2 min, and cooled at room temperature for 5 min. After adding 5 × Apollon buffer, samples were transferred to a clear half-area 96-well plates (Corning). pNPP was added, and absorbance at 405 nm was measured for 4 h using a TECAN Infinite M200 PRO plate reader (TECAN Group) if not stated otherwise. Final concentrations in a typical experiment were 30 μM of the tested oligonucleotide and 1 × Apollon buffer (200 mM KCl, 1 mM ZnCl_2_, and 50 mM HEPES pH 7.4), and 100 μM pNPP. Color production was measured with the following settings: absorbance at 405 (±5) nm, 25 flashes, Z position calculated from one well in the plate. Signal-to-noise ratios were calculated as described in the section ‘Calculation of signal-to-noise ratios’.

### Analysis of phosphorylation using a ligation assay

To measure the extent of 5′ self-phosphorylation, oligonucleotides corresponding to individual sequences from evolved libraries were resuspended in Milli-Q water, heated at 65°C for 2 min, and cooled at room temperature for 5 min. 5 × Apollon buffer and pNPP were then added. Final concentrations in a typical phosphorylation reaction were 1 μM of the tested oligonucleotide, 1 × Apollon buffer (200 mM KCl, 1 mM ZnCl_2_, and 50 mM HEPES pH 7.4), and 1 mM pNPP unless stated otherwise. Reactions were incubated for various times at room temperature and stopped by the addition of EDTA to a concentration of 25 mM. Oligonucleotides were then purified by ethanol precipitation, and reacted oligonucleotides (now containing a 5′ phosphate) were ligated to a short oligonucleotide as described in the section ‘Initial selection’. Reacted and unreacted molecules were separated by 6% denaturing PAGE. DNA was visualized by staining with GelRed using the protocol recommended by the manufacturer. Gels were scanned using a Typhoon laser scanner and the percentage of reacted and unreacted molecules was quantified using ImageQuant TL software.

### Calculation of signal to noise ratios

Signal-to-noise ratios were defined as the absorbance of a sample at 405 nm in the presence of deoxyribozyme divided by the absorbance at 405 nm in the absence of the deoxyribozyme. The background signal was defined as the absorbance at 405 nm of 1 × Apollon buffer (200 mM KCl, 1 mM ZnCl_2_, and 50 mM HEPES, pH 7.4), and was subtracted before calculating signal-to-noise ratios.

### Optimization of reaction conditions

To maximize color production, we performed reactions over a range of conditions using Apollon 2 (Supplementary Table S1). Optimal pNPP, KCl, ZnCl_2_ and HEPES concentrations were determined by titration. We also determined the optimal pH and temperature, and tested the effects of different monovalent metal ions, divalent metal ions, and buffering agents on deoxyribozyme activity. Activity was measured by analysis of color production (using a plate reader assay) and self-phosphorylation (using a ligation assay).

### Kinetic measurements and analysis

Kinetic measurements were performed using Apollon 1 and Apollon 2 (Supplementary Table S1), and reactions were analyzed using a ligation assay. Deoxyribozyme was mixed with Milli-Q water, heated at 65°C for 2 min, and cooled at room temperature for 5 min. 5 × Apollon buffer and pNPP were then added. Final concentrations were 1 μM deoxyribozyme, 1 × Apollon buffer (200 mM KCl, 1 mM ZnCl_2_ and 50 mM HEPES pH 7.4), and 1 μM to 1.5 mM pNPP. Reactions were incubated for specific times at room temperature and stopped by adding EDTA to a concentration of 25 mM. Reactions were stopped at time points that corresponded to the linear phase of the reaction. After ethanol precipitation, reacted deoxyribozymes (containing a 5′ phosphate) were ligated to a short oligonucleotide as described in the section ‘Initial Selection’. Reacted (ligated) molecules were separated from unreacted (unligated) molecules by 6% denaturing PAGE. DNA was visualized by staining with GelRed using the protocol recommended by the manufacturer and gels were scanned using a Typhoon laser scanner. The percentage of reacted and unreacted deoxyribozyme was quantified using ImageQuant TL software. *k*_cat_ and *K*_m_ values were obtained using Prism 9 software. Curves were fitted using the equation (1) to obtain *K*_m_ and with the Equation (2) to obtain *k*_cat_.


(1) \begin{eqnarray*}{{V}_0} = \frac{{{{V}_{{\mathrm{max}}}}\left[ S \right]}}{{{{K}_{\mathrm{m}}} + \left[ S \right]}}\end{eqnarray*}

(2) \begin{eqnarray*}{{k}_{{\mathrm{cat}}}} = \frac{{{{V}_{{\mathrm{max}}}}}}{{\left[ E \right]}}\end{eqnarray*}

### Isothermal titration calorimetry

The thermodynamics of pNPP binding to 5′ phosphorylated Apollon 2 were monitored at 25°C using a VP-ITC microcalorimeter (MicroCal Inc./Malvern Instruments Ltd., UK). Reactant solutions were prepared in a buffer containing 200 mM KCl, 1 mM ZnCl_2_, and 50 mM HEPES pH 7.4. The exact concentration of the HPLC-purified DNA used in these experiments was determined using a NanoDrop100 spectrophotometer. Typically, 9 μl of 1.3 mM pNPP were injected stepwise into a sample cell containing 1.43 ml of 101.3 μM Apollon 2 until saturation was achieved. Experimental titrations were accompanied by corresponding control experiments in which pNPP was injected into buffer that did not contain Apollon 2. Thermodynamic parameters were determined by MicroCal software implemented in Origin 7.0 (MicroCal Inc./Malvern Instruments Ltd, UK).

### Differential scanning calorimetry

Thermal denaturation experiments of Apollon 2 and 5′ phosphorylated Apollon 2 in the presence of 4-nitrophenyl (pNP) or zinc were performed using a high precision VP-DSC differential scanning calorimeter (MicroCal, GE Healthcare, Northampton, MA, USA). HPLC-purified Apollon 2 or 5′ phosphorylated Apollon 2 was annealed in 1 × Apollon buffer (200 mM KCl, 1 mM ZnCl_2_, and 50 mM HEPES pH 7.4), and in same samples zinc was omitted from the buffer. The exact concentration of deoxyribozyme was determined using a NanoDrop100 spectrophotometer and adjusted to 30 μM, while the concentration of pNP was 60 μM. The DNA sample and the buffer reference solutions were degassed by stirring under vacuum and carefully loaded into the calorimeter cells in order to avoid bubble formation. Solutions were heated from 10°C to 90°C at a rate of 1°C/min. Denaturation profiles were superimposed after baseline correction using MicroCal Origin software. The reversibility of heat denaturations was monitored by repeating experiments with already heated samples cooled to 10°C, with additional scans performed after 15 min of thermostatting.

### Next generation sequencing and data analysis

All libraries were sequenced by Eurofins Genomics using amplicon paired-end sequencing runs. Raw reads were processed using a pipeline consisting of adaptor trimming (cutadapt v1.18) (18), read merging (fastq-join v1.3.1) (19), unifying of read orientation (fastx barcode splitter), primer clipping (cutadapt v1.18), length filtering (cutadapt v1.18), and counting of unique sequences (bash). All further analysis was performed using in-house python scripts (https://github.com/Jardic/Apollon_selection_analysis/tree/main).

### Oligonucleotide detection using an engineered version of Apollon

Five different Apollon oligonucleotide sensors were designed (Supplementary Table S1). Each detected a different target oligonucleotide. To detect the presence of a specific target oligonucleotide, the sensor was mixed with the target in water, heated at 98°C for 2 min, and immediately cooled on ice for 5 min. 5 × Apollon buffer was then added. Samples were transferred to a clear half-area 96-well plate (Corning), and pNPP was added. Final concentrations were 50 μM of the oligonucleotide sensor, 100 μM of the target oligonucleotide, 1 × Apollon buffer (200 mM KCl, 1 mM ZnCl_2_, and 50 mM HEPES pH 7.4), and 100 μM pNPP. After incubating for 24 h at room temperature, the absorbance at 405 nm was measured using a TECAN Infinite M200 PRO plate reader. Analysis of color production was performed as described in the section ‘Calculation of signal-to-noise ratios.’

### Ribonuclease detection using a covalently locked version of Apollon

The Apollon ribonuclease sensor was mixed with MilliQ water, heated at 65°C for 2 min, and cooled at room temperature for 5 min. Then 5 × Apollon buffer (1 M KCl, 250 mM HEPES pH 7.4, 5 mM ZnCl_2_) was added. Samples were transferred to a clear half-area 96-well plate (Corning), and pNPP and RNase A (Thermo Fisher Scientific) were added. The reaction mixture was incubated for 4 h at room temperature. Final conditions were 30 μM of the ribonuclease sensor, 300 nM RNase A, 1 × Apollon buffer (200 mM KCl, 1 mM ZnCl_2_, and 50 mM HEPES pH 7.4), and 100 μM pNPP in a final volume of 100 μl if not stated otherwise. The absorbance at 405 nm was measured during the 4 h incubation using a TECAN Infinite M200 PRO plate reader. Analysis of color production was performed as described in the section ‘Calculation of signal-to-noise ratios.’

### DNase I and Exonuclease I inhibitor detection using Apollon

Apollon 2 was mixed with Milli-Q water, 10 × DNase I buffer or 10 × Exonuclease I buffer, and either DNase I or Exonuclease I in a volume of 20 μl. Final concentrations were 150 μM Apollon, 2.5 μM DNase I, and 1 × DNase I buffer (10 mM Tris–HCl pH 7.5, 2.5 mM MgCl_2_ and 0.1 mM CaCl_2_) for detection of DNase I, and 150 μM Apollon 2, 60 U Exonuclease I, and 1 × Exonuclease I buffer (67 mM glycine–KOH pH 9.5, 6.7 mM MgCl_2_, and 1 mM DTT) for detection of Exonuclease I. After an incubation at 37°C for 30 min to allow DNase I or Exonuclese I to cleave and inactivate Apollon 2, 80 μl of 1 × Apollon reaction mixture (200 mM KCl, 1.25 mM ZnCl_2_, 50 mM HEPES pH 7.4, and 125 μM pNPP) was added. Samples were transferred to clear half-area 96-well plate (Corning). The reaction mixture was incubated at room temperature for 4 h, and the absorbance at 405 nm was then measured using a TECAN Infinite M200 PRO plate reader. Analysis of color production was performed as described in the section ‘Calculation of signal-to-noise ratios’. 1 mM ZnCl_2_ was used as an inhibitor of DNase I and Exonuclease I, and was mixed with these enzymes before they were added to the premix reaction.

### Multiple turnover reaction of Apollon

The Apollon constructs used for multiple turnover reactions (Supplementary Table S1) were mixed with Milli-Q water, heated at 65°C for 2 min, and cooled at room temperature for 5 min. 5 × Apollon buffer (1 M KCl, 5 mM ZnCl_2_ and 250 mM HEPES pH 7.4) was then added. Samples were transferred to a clear half-area 96-well plate (Corning), and pNPP was added. Final conditions were 250 μM of the 5′ (substrate) strand, 5 μM of the 3′ (enzyme) strand, 1 × Apollon buffer (200 mM KCl, 1 mM ZnCl_2_, 50 mM HEPES pH 7.4), and 1 mM pNPP in a volume of 100 μl. The reaction mixture was incubated for 7 days at room temperature. Every 4 h the absorbance at 405 nm was measured using a TECAN Infinite M200 PRO plate reader. Analysis of color production was performed as described in the section ‘Calculation of signal-to-noise ratios’. Turnovers were calculated relative to the absorbance produced by 5 μM Apollon 2 over 7 days.

### NMR experiments

HPLC-purified DNA was purchased from GENERI BIOTECH s.r.o. DNA was resuspended in Milli-Q water, heated at 65°C for 2 min, cooled at room temperature for 5 min, and 5 × Apollon buffer was added. Concentrations at this point were 30 μM DNA, 200 mM KCl, 1 mM ZnCl_2_ and 50 mM HEPES pH 7.4. Samples were concentrated to 500 μM DNA using Ultra-Amicon Centrifugal Filter Units (cutoff 3 kDa), and a 1.5 molar excess of pNPP, D_2_O and DSS were added. Final concentrations were 500 μM DNA, 200 mM KCl, 1 mM ZnCl_2_, 50 mM HEPES pH 7.4, 750 μM pNPP, 10% (v/v) D_2_O, and a trace amount of DSS if not stated otherwise. NMR experiments were performed on a Bruker Avance III HD 850 MHz system equipped with an inverse triple-resonance cryo-probe. Spectral analyses were performed using TOPSPIN (Bruker) and MestReNova software.

## Results and discussion

### Discovery of deoxyribozymes that react with a colorimetric substrate

**Figure 1.**
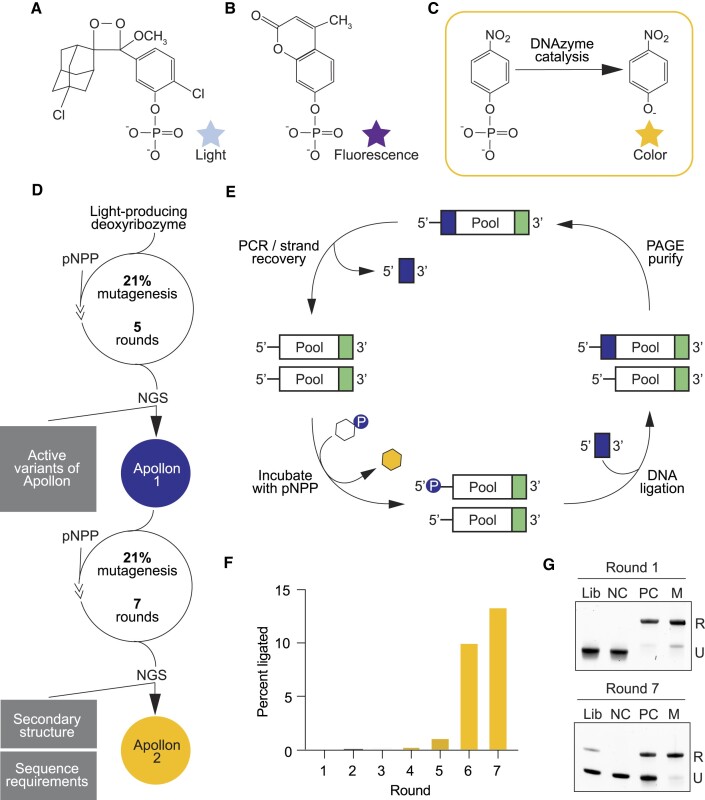
Discovery of deoxyribozymes that react with the colorimetric substrate pNPP. (**A**) Chemical structure of CDP-Star, the chemiluminescent substrate used by Supernova (a deoxyribozyme previously discovered in our group). (**B**) Chemical structure of 4-methylumbelliferyl phosphate (4-MUP), the fluorescent substrate used by Aurora (a deoxyribozyme previously discovered in our group). (**C**) Chemical structure of pNPP, the colorimetric substrate used in this study. (**D**) Workflow used to identify Apollon 1 (the minimized catalytic core of the initial isolate of Apollon) and Apollon 2 (an optimized variant isolated from a randomly mutagenized library based on Apollon 1). (**E**) *In vitro* selection protocol to identify deoxyribozymes that phosphorylate themselves in the presence of pNPP. (**F**) Graph showing percent of ligated molecules during the reselection experiment that yielded Apollon 2 from a randomly mutagenized library based on Apollon 1. (**G**) Denaturing PAGE gel showing the library after 1 and 7 rounds of reselection for Apollon 2.

In previous studies we identified deoxyribozymes that produce chemiluminescent (10,11) and fluorescent (12) signals by selecting for DNA molecules that phosphorylate themselves in the presence of substrates that generate light or fluorescence when they are dephosphorylated (Figure 1A, B). Because colorimetric assays do not require specialized equipment and are typically less expensive than chemiluminescent and fluorescent ones, colorimetric deoxyribozymes could also be useful. The starting point for our efforts to identify such deoxyribozymes was the small molecule substrate pNPP (Figure 1C). Dephosphorylation of pNPP yields the yellow product pNP (17), and deoxyribozymes that catalyze this reaction could in principle be used in colorimetric assays. A library was prepared by randomly mutagenizing 85 positions in the H1 variant of Supernova (a chemiluminescent deoxyribozyme previously identified in our group) at a rate of 21% per position (Figure 1D). This yielded a library of 1.7 × 10^14^ molecules. We chose Supernova as the starting point for our library because it catalyzes a phosphoryl transfer reaction using a substrate with some similarities to pNPP (compare Figure 1A and C). Variants that could transfer the phosphate group from pNPP to their 5′ hydroxyl group were tagged by ligation, purified by PAGE, and amplified by PCR (Figure 1E) (10,16). The selection was performed in a buffer containing 200 mM potassium (which we thought would help to shield charge from negatively charged phosphate groups during deoxyribozyme folding) as well as lower concentrations of lead, zinc, and cerium (which promote the nonenzymatic dephosphorylation of CDP-Star (10), and could potentially be used as cofactors by deoxyribozymes that catalyze dephosphorylation of pNPP). After four rounds of selection a faint signal was detected, and after one more round the evolved library was characterized by high-throughput sequencing. 4 393 681 total reads and 15 828 unique sequences were obtained (Supplementary Figure S1). The deoxyribozymes isolated in this initial selection could phosphorylate themselves using pNPP, but were not efficient enough to generate a useful colorimetric signal. For this reason, further optimization was necessary.

### Identification of the catalytic core of the colorimetric deoxyribozyme Apollon

**Figure 2.**
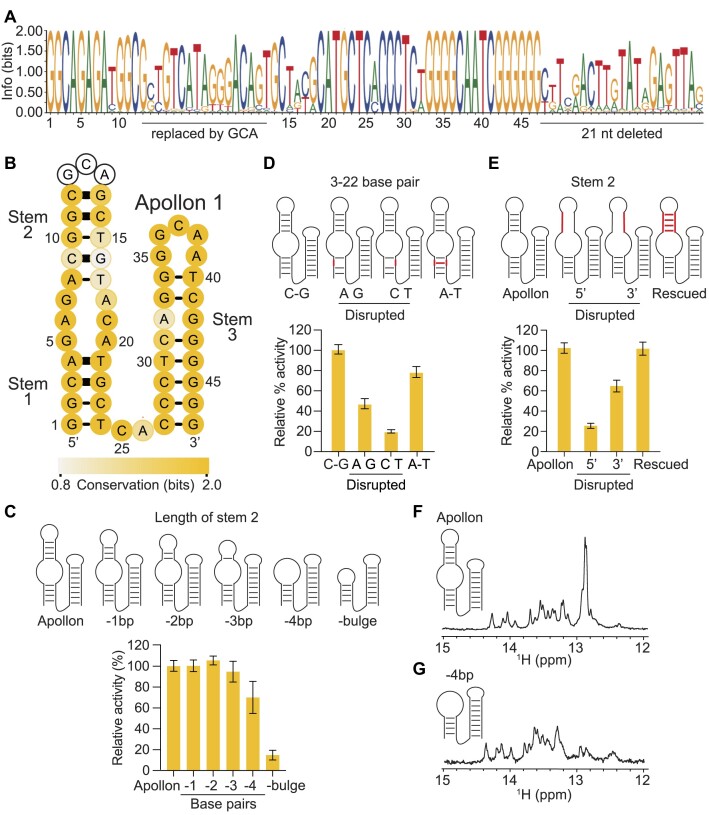
Sequence requirements and secondary structure of Apollon. (**A**) Sequence logo generated from analysis of variants of Apollon using high-throughput sequencing. (**B**) Secondary structure model of Apollon 1. Base pairs are shown using thin black lines, base pairs supported by covariation analysis are shown using thick black lines, and the degree of conservation at each position is indicated by yellow shading. (**C**) Deletion analysis of Stem 2. (**D**) Double mutant cycle showing that positions 3 and 22 interact in a manner consistent with base pairing. (**E**) Mutant cycle showing the importance of Stem 2 for Apollon function. (**F**) Proton NMR spectra of Apollon 2 showing signals consistent with base pairs in the presence of pNPP and ZnCl_2_. (**G**) Proton NMR spectra of a catalytically active mutant of Apollon 2 in which Stem 2 has been deleted.

The deoxyribozyme with the highest read number from the initial selection (corresponding to 78.5% of the total reads; Supplementary Figure S1) was chosen for further characterization. We named this sequence Apollon 1 full-length, and the catalytic motif itself Apollon. Apollon 1 full-length appeared to be distinct from the starting deoxyribozyme Supernova, as did most deoxyribozymes isolated in this selection (Supplementary Figure S2). A second library was generated by randomly mutagenizing the sequence of Apollon 1 full-length at a rate of 21% per position (Figure 1d), and catalytically active variants were identified by performing a reselection using conditions more stringent than those utilized in the original selection experiment. After four rounds of reselection activity could be detected (Figure 1f), and after three more rounds the library was characterized using both biochemical assays (Figure 1F, G) and high-throughput sequencing. 5 657 508 total reads and 34 493 unique sequences were obtained (Supplementary Figure S1). Analysis of conservation at each position in the deoxyribozyme suggested that positions 1–8 and 34–64 likely form the catalytic core of Apollon 1 full-length, whereas other positions might not be necessary for activity (Figure 2a). The activity of a 39-nucleotide long construct which only contained positions 1–8 and 34–64 was confirmed using a spectroscopic assay (Supplementary Figure S3). However, addition analysis revealed that the activity of a 50-nucleotide long deoxyribozyme in which the region between positions 9 and 33 was extended with a stem was about 20% higher than that of the catalytic core alone (Figures 2B, C and Supplementary Figure S3). For this reason, the 50-nucleotide variant (which we named Apollon 2) was used for most of the experiments described here.

### Sequence requirements and secondary structure of Apollon

Our next goal was to develop a secondary structure model of Apollon. Such models can often be identified by searching for covariations (pairs of positions that change in a correlated way) consistent with base pairing in datasets of variants of a functional nucleic acid motif (9,20–22). In the case of Apollon, however, this approach was limited by high levels of conservation at most positions in the catalytic core. Despite this, we were able to develop a secondary structure model by combining results from comparative analysis with information from double mutant cycles (in which putative base pairs are disrupted by separately mutating each of the two positions in the pair and restored by combining these two deleterious mutations) and proton NMR experiments. Our model contains three helical regions, called Stem 1, Stem 2 and Stem 3, and several conserved loops and bulges (Figure 2B and Supplementary Figure S4a). Stem 1 is well-supported by both covariation analysis and double-mutant cycles (Figure 2D). Stem 2 is less important: individual base pairs could be deleted without reducing activity (Figure 2C), and a variant in which Stem 2 was deleted was only slightly less active than a variant in which it was present (Figure 2C and Supplementary Figure S3). However, mutant cycles in which multiple base pairs in Stem 2 were disrupted by mutations in the 5′ half of the helix, disrupted by mutations in the 3′ half of the helix, or restored by mutations in both the 5′ and 3′ halves of the helix demonstrated that formation of this stem is important in some sequence backgrounds (Figure 2E). Evidence for Stem 3 was less conclusive. Because this part of the sequence was almost invariant (Figure 2A, B), no covariations were observed. However, several additional lines of evidence supported Stem 3 as well as other aspects of the proposed structure. First, most active variants (and all with a read number of 10 000 or greater) had the potential to form at least seven of eight base pairs in Stem 3 (Supplementary Figure S4), and this degree of pairing was unlikely to occur by chance in randomly chosen subsets of sequences in the starting library (Supplementary Figure S4b). Second, the proton NMR spectrum of Apollon 2 contains ∼20 distinct signals consistent with base pairs (23) (Figure 2F and Supplementary Figure S5). This spectrum is consistent with our secondary structure model, which contains 12 canonical base pairs (each of which would be expected to yield one signal in the 12 ppm to 15 ppm range of the spectrum) and four G–T or T–G pairs (each of which would be expected to yield two signals in this part of the spectrum due to the two tautomeric forms of guanine and thymine). Deletion of the four canonical base pairs in Stem 2 of Apollon 2 yielded a catalytically active variant with ∼16 distinct signals (Figure 2G), which provided additional support for the proposed structure. Although a G-quadruplex could also in principle form in a G-rich region like that of Stem 3, the spectrum does not contain signals in the 10 ppm to 12 ppm range (Figures 2F, G), and is therefore not consistent with such a structure (23). The sequence of Apollon 2 also contains two stretches of at least three consecutive pyrimidines and three stretches of at least three consecutive purines, and therefore could also potentially form triple helical structures like that proposed to occur in Supernova (10). However, such structures are not supported by either comparative sequence analysis or triple-mutant cycles (Supplementary Figure S6). Taken together, these experiments suggest that Apollon forms a secondary structure consisting of three stems, one with a highly conserved primary sequence.

### Apollon requires multiple zinc ions for function

**Figure 3.**
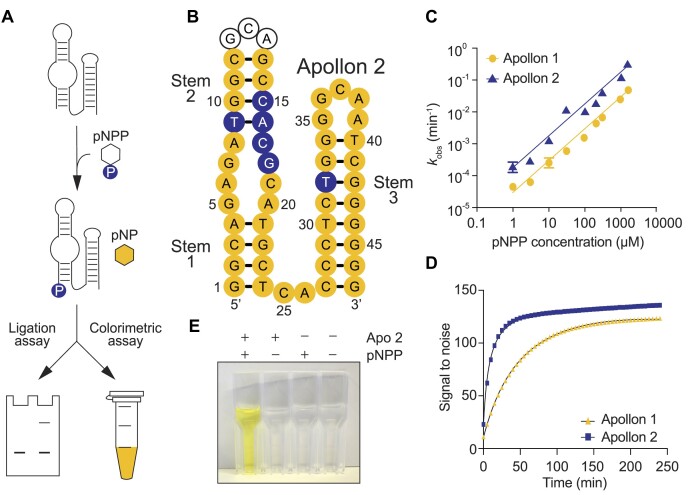
An optimized version of Apollon produces a colorimetric product that can be visualized by eye. (**A**) Assay to measure deoxyribozyme self-phosphorylation (ligation assay) and production of a yellow product (colorimetric assay). (**B**) Secondary structure model of Apollon 2. Positions that differ from Apollon 1 are shown in blue. (**C**) Catalytic activity of Apollon 1 and 2 over a range of pNPP concentrations as measured using a ligation assay. (**D**) Colorimetric reaction of Apollon 1 and Apollon 2 analyzed using a plate reader. (**E**) Example of a reaction that can be visualized by eye.

After identifying the minimized catalytic core and developing a secondary structure model, we next characterized the buffer requirements of Apollon using both ligation and spectroscopic assays (Figure 3A). The effects of buffer conditions on deoxyribozyme folding were also monitored by analysis of imino proton signals in 1D ^1^H NMR spectra. Because these signals (which occur between 10 ppm and 15 ppm) are separated from those of other protons in nucleic acids, they can be used to monitor formation of nucleic acid secondary structures. These experiments showed that, of the four ions present in the selection buffer (zinc, lead, cerium, and potassium), only zinc is required for activity (Supplementary Figure S7). Potassium could be replaced by other monovalent metal ions such as lithium, sodium, rubidium, or cesium without affecting catalytic function (Supplementary Figure S8), and activity was similar over a range of potassium concentrations (Supplementary Figure S9). In contrast, zinc could not be replaced by any of the other divalent metal ions we tested (Supplementary Figure S10). Titration experiments showed that zinc affects catalytic activity in a highly cooperative way, with Hill coefficients ranging between 2.7 and 3.8 depending on the assay (Supplementary Figures S11 and S12). This suggests that multiple zinc ions are required for folding and/or activity, although determining the exact number is not possible from these data. High levels of cooperativity were also observed when titrations were performed in the presence of either 10 mM MgCl_2_ or 1 mM [Co (NH_3_)_6_]Cl_3_ indicating that these zinc binding sites are highly specific (Supplementary Figure S13). Activity did not specifically require HEPES (Supplementary Figure S14), but was highly dependent on pH, and only observed between pH 7 and pH 8 (Supplementary Figure S15). Maximal reaction rates were observed between 25°C and 55°C, but decreased rapidly at higher or lower temperatures (Supplementary Figure S16). These buffer requirements are similar to those of Supernova (10,11) and Aurora (12). A particularly interesting parallel is that each of these three deoxyribozymes requires zinc ions for activity when in principle they could have required any other combination of the four metal ions present in the selection buffer. This suggests that zinc plays an important role in deoxyribozymes that promote phosphoryl transfer reactions using monophosphorylated substrates, as has been previously noted for deoxyribozymes that promote DNA cleavage reactions (24–26). In a more general sense, these results highlight the extent to which the presence of specific metal ions during a selection can increase the likelihood of obtaining nucleic acid enzymes with specific functions (27).

### An optimized version of Apollon generates a yellow product that can be seen by eye

After identifying optimal conditions for the reaction, we next characterized the catalytic parameters of Apollon 1 (the initial isolate of Apollon) (Figure 2B) and Apollon 2 (the variant with the highest read number after reselection; Figure 3B). It was not possible to saturate the binding sites of these deoxyribozymes even at concentrations of pNPP as high as 1.5 mM, indicating that Apollon has a low affinity for its substrate (Figure 3C). This is perhaps not surprising given the small size of pNPP. However, both the catalytic efficiency (*k*_cat_/K_M_) of Apollon 2 of about 200 M^−1^ min^−1^ and the maximum *k*_obs_ of 0.3 min^−1^ were comparable to those of Supernova (10,11) and Aurora (12). The catalytic efficiency (*k*_cat_/K_M_) of Apollon 2 was 10-fold higher than that of Apollon 1 (Figure 3C). It also generated a stronger colorimetric signal, with a signal-to-noise ratio of approximately 150 (Figure 3D and Supplementary Figure S17) and an initial rate 1020-fold higher than that of the nonenzymatic reaction measured under the same conditions (Supplementary Figure S18). This signal-to-noise ratio was only slightly lower than the ratio obtained by comparing the absorbance of a solution of synthetic pNP with that of pNPP (Supplementary Figure S19). The signal could also be detected by eye (Figure 3E). Apollon 2 contained six mutations in the catalytic core relative to Apollon 1. Five of these occurred in or near the bulge between Stem 1 and Stem 2 (Figure 3B), indicating that this part of the deoxyribozyme is particularly important for catalytic function. Taken together, these experiments provide information about mutations that enhance the catalytic activity of Apollon. They also demonstrate that our approach can be used to identify colorimetric deoxyribozymes that generate signals strong enough to be seen by eye.

### Apollon makes strong contacts with the phosphate group in the substrate

**Figure 4.**
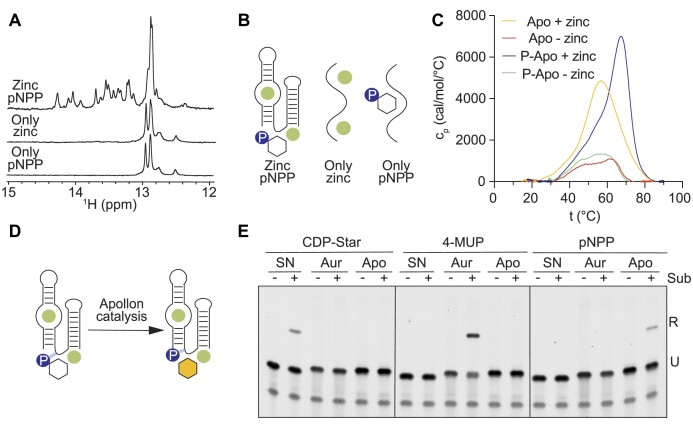
Apollon makes contacts with the phosphate group in the product. (**A**) Proton NMR spectra of Apollon 2 showing signals consistent with base pairs in the presence of pNPP and ZnCl_2_. (**B**) Apollon requires zinc and a phosphate on either the substrate or its own 5′-hydroxyl group for folding. (**C**) Denaturation profiles of Apollon 2 and 5′ phosphorylated Apollon 2 in the absence and presence of zinc. (**D**) Aurora makes contacts with the phosphate group in the product. (**E**) Catalytic activity of Supernova (SN), Aurora 2 (Aur) and Apollon 2 (Apo) in the presence of CDP-Star, 4-MUP and pNPP measured using the ligation assay.

We next investigated how Apollon recognizes its substrate. Isothermal titration calorimetry provided an important clue. No binding of pNPP to Apollon was detected when a substrate titration was performed using reacted (5′ phosphorylated) deoxyribozyme (Supplementary Figure S20), suggesting that this phosphate remains in or near the active site even after the reaction. NMR experiments also showed that folding requires the presence of both zinc and a phosphate group on either the substrate or the 5′ hydroxyl group of Apollon (Figures 4A, B and Supplementary Figure S21a). We note that a base pair must be stable on a milisecond time-scale in order to provide a detectable imino proton signal. Less stable base pairs yield broader peaks, which are eventually lost in spectral noise. Similarly, the existence of multiple deoxyribozyme conformations that exchange on milisecond time-scales result in undetectable imino proton signals. Therefore, the lack of imino proton signals in these spectra suggests the the structure of Apollon is unstable, heterogeneous, or both in the absence of zinc and a phosphate on either the substrate or the 5′ hydroxyl of Apollon. Additional evidence that Apollon makes contacts with the phosphate group in the product came from differential scanning calorimetry. This showed that the melting temperature of the reacted form of the Apollon (which contains a 5′ phosphate) is 7°C higher than that of the unreacted form (which contains a 5′ hydroxyl group) (Figure 4C). This difference in melting temperature was only observed when folding was performed in the presence of zinc (Figure 4C and Supplementary Figure S21b), highlighting its important role in deoxyribozyme folding and function (Figure 4D). These observations are consistent with a model in which Apollon makes important contacts with the phosphate group in pNPP (Figure 4D). However, other characteristics of the substrate-binding pocket must also be important, since Apollon is relatively specific for its substrate. For example, the substrate specificity of Apollon is orthogonal to that of both Supernova and Aurora (Figure 4E and Supplementary Figure S22), perhaps due to steric clashes with the larger substrates used by these deoxyribozymes (compare Figure 1A–C). Furthermore, Apollon does not generate a ligated product when reactions are performed in the absence of pNPP, indicating that it cannot phosphorylate itself using the ATP in the ligation buffer (Supplementary Figure S23). Taken together, these results indicate that Apollon requires a phosphate group in the substrate for folding, also makes contacts with the phosphate in the product, and forms a relatively specific binding site for pNPP. They also provide additional evidence that zinc helps to stabilize the overall fold of the deoxyribozyme, including the structure of the active site.

### Apollon catalyzes multiple turnover phosphorylation

**Figure 5.**
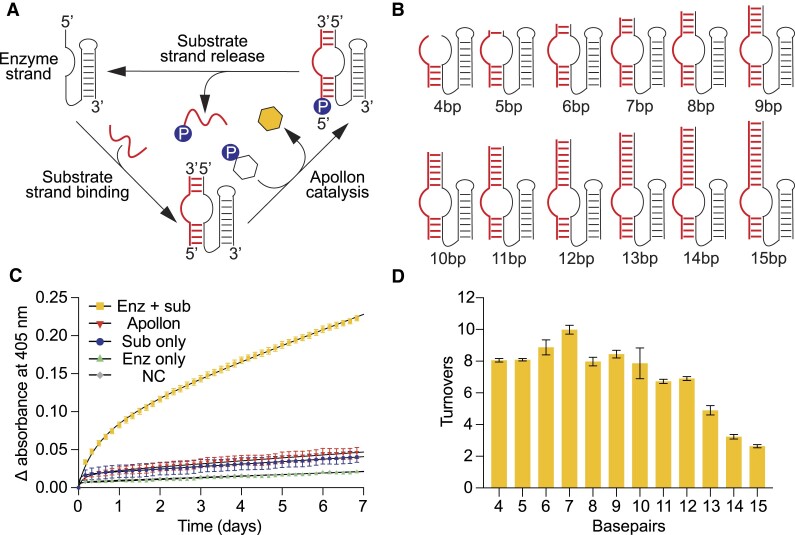
Multiple turnover reaction of Apollon. (**A**) Overview of the multiple turnover reaction. (**B**) Schematic representation of constructs with substrate binding helices of different lengths. (**C**) Colorimetric assay comparing the signal generated by a multiple-turnover construct containing a substrate binding helix of seven base pairs with that generated by a unimolecular single-turnover construct. NC shows the signal generated by pNPP in buffer alone. (**D**) Graph showing the number of turnovers catalyzed by different constructs in seven-day incubations.

A limitation of conventional selection experiments is that catalytic molecules must modify themselves in order to survive the selection step. For this reason, many ribozymes and deoxyribozymes identified in such experiments catalyze single turnover reactions. A common strategy to overcome this limitation is to divide a catalytic motif into a substrate strand (which contains the reaction site) and an enzyme strand (which contains the rest of the motif) (28–30). In the presence of an excess of the substrate strand, a single enzyme strand can bind, react with, and release multiple substrate strands (Figure 5A). Designing such a construct is greatly facilitated by knowledge of the secondary structure of the motif. In the case of Apollon, comparative sequence analysis indicates that the 5′ end forms a hairpin in which the loop nucleotides are not conserved. This suggested that it might be possible to generate a bimolecular version of Apollon by deleting the loop in the 5′ hairpin. This turned out to be the case, and the catalytic activity of the bimolecular construct (tested using a 1:1 ratio of the enzyme strand to the substrate strand) was similar to that of the unimolecular one. To investigate the extent to which this variant could promote multiple turnover phosphorylation, a series of bimolecular constructs were generated in which the number of base pairs in the helix formed between the enzyme strand and the substrate strand were varied (Figure 5B). When incubated in the presence of an excess of the substrate strand, an enzyme strand generated a stronger colorimetric signal than a unimolecular deoxyribozyme incubated at the same concentration (Figure 5C), suggesting that it promotes multiple turnover catalysis. We also investigated the effect of adding or deleting base pairs between the enzyme strand and substrate strand on the efficiency of the multiple turnover reaction. Constructs with short recognition helices were expected to be limited by substrate strand binding, whereas constructs with long helices were expected to be instead limited by substrate strand release (30). In the case of Apollon, the optimal length of the helix between the enzyme strand and substrate strand turned out to be seven base pairs, and this construct catalyzed approximately ten turnovers when the reaction was followed for seven days (Figure 5D). These results show that Apollon can catalyze multiple turnover phosphorylation, and that this can enhance the strength of the colorimetric signal.

### Apollon can detect a range of inputs in homogenous assays

**Figure 6.**
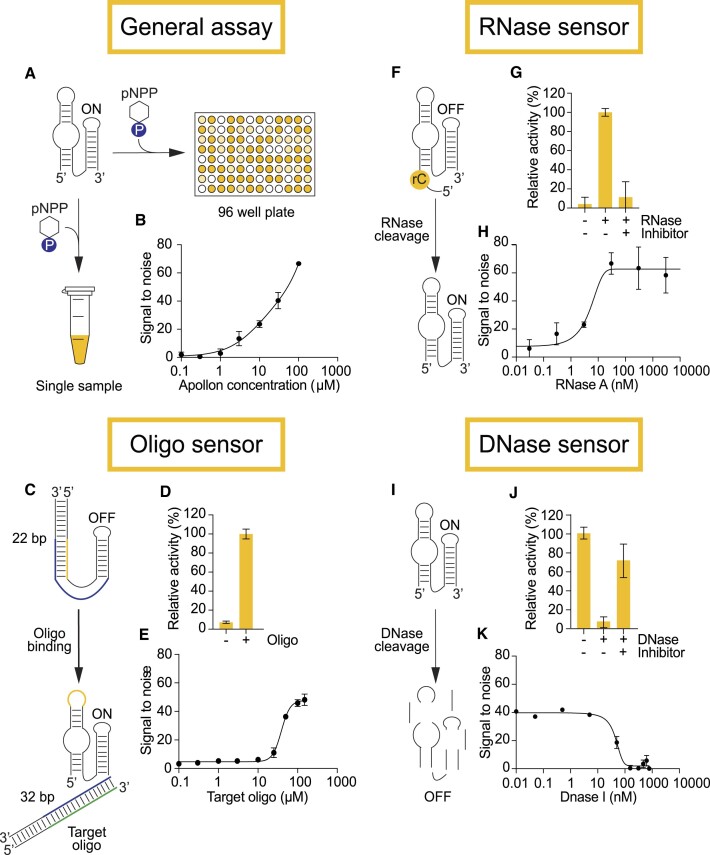
Diverse architectures of Apollon sensors. (**A**) Workflow of the colorimetric reaction of Apollon. (**B**) Sensitivity of the reaction. (**C**) Architecture of an Apollon sensor that detects oligonucleotides. (**D**) Activation of this oligonucleotide sensor by a target oligonucleotide. (**E**) Sensitivity of the oligonucleotide sensor. (**F**) Architecture of an Apollon sensor that detects RNases. (**G**) The Apollon RNase sensor is activated by RNase A, but not when an RNase inhibitor is present. (**H**) Sensitivity of the RNase sensor. (**I**) Architecture of an Apollon sensor that detects DNases. (**J**) DNase I inactivates the Apollon nuclease sensor, but not when a DNase inhibitor is present. (**K**) Sensitivity of the nuclease sensor.

Because the workflow of the colorimetric reaction catalyzed by Apollon contains few steps and requires little experimental manipulation (Figure 6A, B), it could be a useful tool in homogenous assays (here used to describe assays that can be performed in a single tube without the need for washes or purifications) (31). In this regard, the ability to construct variants of Apollon that only generate a signal in the presence of a ligand of interest would be particularly useful (32,33), especially if such variants can be generated rapidly (34). To show that this is feasible, we developed three different sensor architectures based on Apollon. The first type of sensor only generated a signal in the presence of oligonucleotides with specific sequences (Figure 6C). Such sensors could in principle be useful for applications such as diagnostics and molecular computing (35). To make this sensor, part of the sequence of the target oligonucleotide was added to the loop at the end of the Stem 2 helix, and the full reverse complement of the target was added to the 3′ end of Apollon. Because these two insertions are complementary, they were expected to interact by base pairing, which should interfere with formation of Stems 1 and 2 in Apollon and inhibit deoxyribozyme activity. Consistent with our model, this construct was catalytically inactive (Figure 6D). In the presence of the target oligonucleotide, however, base pairing between the target and the 3′ end of the deoxyribozyme prevented this inhibitory interaction from occurring and restored catalytic activity (Figure 6D). Our sensor generated a yellow product only in the presence of a target oligonucleotide with a signal-to-noise ratio (defined here as the signal in the presence of the target oligonucleotide divided by the signal in the absence of the target oligonucleotide) of 13.7-fold (Figure 6D). It was also programmable (Supplementary Figure S24). A limitation of this architecture was low sensitivity, with a limit of detection of approximately 20 μM (Figure 6E). In addition, some off-target activation was observed (Supplementary Figure S24). These observations suggest that our proof-of-principle sensor would benefit from further optimization. This could potentially be achieved by reselection (32–34,36,37).

To address some of these limitations, a second architecture was developed that could be used to detect ribonuclease activity (Figure 6F). To construct this sensor, a short DNA spacer with a single RNA base at its 3′ end was covalently attached to the 5′ hydroxyl group of Apollon. Because Apollon transfers the phosphate group of pNPP to its 5′ hydroxyl group, this modification inactivated the deoxyribozyme. However, when incubated in the presence of a ribonuclease that generates products with 3′ phosphate (or 2′-3′ cyclic phosphate) and 5′ hydroxyl termini, the single RNA linkage in this sensor was cleaved and catalytic activity was restored. Because protein ribonucleases typically promote multiple turnover reactions, an advantage of this design is that it results in amplification of the signal. Our ribonuclease sensor generated a yellow product with a signal-to-noise (defined here as the signal in the presence of the ribonuclease divided by the signal in the absence of the ribonuclease) of 10.6-fold (Figure 6G and Supplementary Figure S25) and a detection limit of 10 nM for RNase A (Figure 6H). This is 100-fold to 1000-fold lower than the detection limit of Apollon itself (Figure 6B), and highlights the importance of signal amplification. Importantly, these sensors can distinguish between reactions that contained RNase inhibitors and those that do not (Figure 6g and Supplementary Figure S25), indicating that they could be used for high-throughput screening. RNase inhibitors that target bacterial RNases such as RNase E and RNase P or viral RNases such as RNase H, Nsp14, and Nsp15 could be useful as lead compounds (38), and in an accompanying manuscript we showed that it is possible to identify inhibitors of the Nsp15 RNase from SARS-CoV-2 using a deoxyribozyme-based assay (12). RNase inhibitors are also useful as RNA-stabilizing reagents and as tools to dissect catalytic mechanisms (39).

A third type of sensor used unmodified Apollon to detect nucleases that cleave DNA (Figure 6I). When incubated in the absence of nuclease, this sensor was expected to generate a colorimetric signal. In the presence of nuclease, however, the sensor should be degraded, which will result in a reduction of the signal. We tested this design using the nucleases DNase I and Exonuclease I. Signal-to-noise ratios of 20.7-fold were achieved (Figure 6J and Supplementary Figure S26), with detection limits between 10 and 100 nanomolar (Figure 6K). These sensors can also distinguish between reactions that contained DNase or exonuclease inhibitors and those that do not, suggesting that could be used to identify inhibitors in high-throughput screens. Such inhibitors could be used to target DNases that play a role in bacterial infections such as Sda1 (40), to target nucleases associated with inflammatory diseases such as DNase γ (41,42), and to better understand DNase catalytic mechanisms. Taken together, these experiments show that Apollon can be used to sense a wide range of inputs in homogenous assays.

### Advantages of Apollon compared to other methods of generating colorimetric signals

One inspiration for the approach used in this study was the protein enzyme alkaline phosphatase. Like Apollon, alkaline phosphatase can react with pNPP to generate a yellow product. Advantages of alkaline phosphatase include its remarkable catalytic efficiency (*k*_cat_/K_M_), which approaches the diffusion-controlled limit (43), and the ability to catalyze multiple turnover reactions. On the other hand, alkaline phosphatase is more expensive to manufacture and less stable than Apollon. It is also less specific with regard to its substrate, which means that it is not suitable for multiplex assays, and more difficult to modify by rational design or artificial evolution, which is a significant limitation with respect to development of homogenous assays. A second inspiration was a peroxidase deoxyribozyme discovered by the Sen group that converts the substrate ABTS into a colorimetric product in the presence of hydrogen peroxidase and hemin (44,45). Like Apollon, this deoxyribozyme is small, inexpensive to synthesize, and uses a widely available substrate. It is active over an even wider range of conditions than Apollon, including at pH 3 (46) and at 95°C (47). A drawback is that the reaction requires hydrogen peroxide. This is not compatible with many assays and can also damage the hemin cofactor. When comparing Apollon to these other enzymes, we note that each offers unique possibilities with respect to sensor development. For example, the architecture of the RNase sensor described in this study could not be easily adapted to either alkaline phosphatase (because an RNA oligonucleotide covalently linked to the serine nucleophile would not be a substrate for most RNases) or a peroxidase deoxyribozyme (because this motif does not use its 5′ or 3′ hydroxyl group as a nucleophile). From this perspective, we suggest that Apollon should be thought of as a complementary tool as compared to these other enzymes. In cases in which more sensitive assays can be developed using protein enzymes such as alkaline phosphatase, Apollon also potentially offers advantages due to its low cost. For example, a high-throughput screen could initially be performed using Apollon to narrow down the number of potential inhibitors in a library, and a second, more sensitive but also more expensive screen could then be performed using only these hits to identify the most potent compounds in the library.

## Conclusions

In this study, we developed both single and multiple turnover versions of a self-phosphorylating deoxyribozyme called Apollon that converts the colorless substrate pNPP into the yellow product pNP. We also showed that Apollon can be modified to act as a sensor for a variety of inputs in homogenous assays. The detection limit of the most sensitive of these sensors is in the low nanomolar range, and some could also distinguish between the presence and absence of enzyme inhibitors in samples. Because reactions can be analyzed without specialized equipment, we suggest that Apollon could be useful for applications such as high-throughput screens, field work and point-of-care assays. In a more general sense, this work adds an important new component to the toolkit of functional DNA parts, and highlights the functional diversity of catalytic DNA.

## Supplementary Material

gkae490_Supplemental_File

## Data Availability

The data underlying this article will be shared on reasonable request to the corresponding author.
