# Supplementary material for: Apollon: a deoxyribozyme that generates a yellow product
Source: Nucleic Acids Res. 2024 Jun 13;52(15):9062–75. doi: 10.1093/nar/gkae490 (PMC11347176; doi:10.1093/nar/gkae490)
Supplement: gkae490_Supplemental_File [file gkae490_supplemental_file.docx]

**Apollon: a deoxyribozyme that generates a yellow product**

Martin Volek^1,2^, Jaroslav Kurfürst^1,3^, Milan Kožíšek^1^, Pavel Srb^1^, Václav Veverka^1,4^, and Edward A. Curtis^1*^

^1^Institute of Organic Chemistry and Biochemistry of the Czech Academy of Sciences, Prague 166 10, Czech Republic

^2^Department of Genetics and Microbiology, Faculty of Science, Charles University in Prague, Prague 128 44, Czech Republic

^3^Department of Informatics and Chemistry, University of Chemistry and Technology, Prague 166 28, Czech Republic

^4^Department of Cell Biology, Faculty of Science, Charles University in Prague, Prague 128 44, Czech Republic

Tel: +420 733 169 654 ; Email: curtis@uochb.cas.cz

**SUPPLEMENTARY TABLES**

Supplementary Table 1**.** Sequences of deoxyribozymes and oligonucleotides used in this study.

| **Name** | **Nucleotide sequence** |
| --- | --- |
| Pool1 | GGAAGAGATGGCGACGACACAGGGACGATGCCGAATATCCTCAGTCGCAGGGCCGCAGGGGGGAGTGACTTGGGATGGGGGGTCCACTAATATCTGCCCGATG (underlined NTs were mutagenized at the rate of 21%) |
| FWD1 | ACCGCTCAGGTGTAGTATCA |
| REV1 | CATCGGGCAGATCATTAGTG |
| Splint1 | GTCGCCATCTCTTCCTGATACTACACCTGAGCGGT |
| FWD1r | ACCGCTCAGGTGTAGTATCrA |
| REV1p | pCATCGGGCAGATCATTAGTG |
| Apollon1-full length 85nt | GGCAGAGACGGCGGCGTCATAGGGACAGTGCTGTACATGCTCACCCTCAGGGGCAATCGGGGGGCGTGAACTTATATGGAGTTAC |
| Apollon1-core 50nt | GGCAGAGACGGCGCAGCTGTACATGCTCACCCTCAGGGGCAATCGGGGGG |
| Pool2 | GGCAGAGACGGCGGCGTCATAGGGACAGTGCTGTACATGCTCACCCTCAGGGGCAATCGGGGGGCGTGAACTTATATGGAGTTACTAACACTCTTGACGAGCCGT (underlined NTs were mutagenized at the rate of 21%) |
| REV2 | ACGGCTCGTCAAGAGTGTTA |
| REV2p | pACGGCTCGTCAAGAGTGTTA |
| Apollon2-full length 85nt | GGCAGAGATGGCGCTGTCATAGGGACAGTGCTACGCATGCTCACCCTCTGGGGCAATCGGGGGGCTTCGACTTGTATAGAGTTAG |
| Apollon2-core 64nt | GGCAGAGATGGCGCTGTCATAGGGACAGTGCTACGCATGCTCACCCTCTGGGGCAATCGGGGGG |
| Apollon2-core 50nt  (typically referred  to as Apollon 2) | GGCAGAGATGGCGCAGCCACGCATGCTCACCCTCTGGGGCAATCGGGGGG |
| Apollon2-core 39nt | GGCAGAGACGCATGCTCACCCTCTGGGGCAATCGGGGGG |
| Apollon 2 - 5'disrupted | GGCAGAGAACTGGCAGCCACGCATGCTCACCCTCTGGGGCAATCGGGGGG |
| Apollon 2 - 3'disrupted | GGCAGAGATGGCGCACAGTCGCATGCTCACCCTCTGGGGCAATCGGGGGG |
| Apollon 2 - rescue | GGCAGAGAACTGGCACAGTCGCATGCTCACCCTCTGGGGCAATCGGGGGG |
| Oligo sensor 1 | GGCAGAGATGGCGAAGGTCAATGCCACGCATGCTCACCCTCTGGGGCAATCGGGGGGATTGACCTTCATTGACCTTCGCCATCTCTGCC |
| Oligo sensor 2 | GGCAGAGATGGCGGGCACTGATGCAGCCACGCATGCTCACCCTCTGGGGCAATCGGGGGGATCAGTGCCCAGTGGGCACGGCCATCTCTGCC |
| Oligo sensor 3 | GGCAGAGATGGCGATGATCGGAGCAGCCACGCATGCTCACCCTCTGGGGCAATCGGGGGGTCCGATCATCCGAAGATCAGGCCATCTCTGCC |
| Oligo sensor 4 | GGCAGAGATGGCAAGTAATAGCGCAGCCACGCATGCTCACCCTCTGGGGCAATCGGGGGGGCTATTACTTATCTTTCCGAGCCATCTCTGCC |
| Oligo sensor 5 | GGCAGAGATGGCTAAAAGATAAGCAGCCACGCATGCTCACCCTCTGGGGCAATCGGGGGGTTATCTTTTATTCGTGTGTAGCCATCTCTGCC |
| Target Oligo 1 | GGCAGAGATGGCGAAGGTCAATGAAGGTCAA |
| Target Oligo 2 | GGCAGAGATGGCCGTGCCCACTGGGCACTGAT |
| Target Oligo 3 | GGCAGAGATGGCCTGATCTTCGGATGATCGGA |
| Target Oligo 4 | GGCAGAGATGGCTCGGAAAGATAAGTAATAGC |
| Target Oligo 5 | GGCAGAGATGGCTACACACGAATAAAAGATAA |
| Apollon - RNase A sensor | AAAArCGGCAGAGATGGCGCAGCCACGCATGCTCACCCTCTGGGGCAATCGGGGGG |
| Supernova | GGAAGAAAAAGAATATCCCCAAAAGGGGAGTGACTTGGGATGGGGG |
| MT_substrate strand_4bp | GGCAGAGA |
| MT_enzyme strand_4bp | CGCATGCTCACCCTCTGGGGCAATCGGGGGG |
| MT_substrate strand_5bp | GGCAGAGAT |
| MT_enzyme strand_5bp | ACGCATGCTCACCCTCTGGGGCAATCGGGGGG |
| MT_substrate strand_6bp | GGCAGAGATG |
| MT_enzyme strand_6bp | CACGCATGCTCACCCTCTGGGGCAATCGGGGGG |
| MT_substrate strand_7bp | GGCAGAGATGG |
| MT_enzyme strand_7bp | CCACGCATGCTCACCCTCTGGGGCAATCGGGGGG |
| MT_substrate strand_8bp | GGCAGAGATGGCG |
| MT_enzyme strand_8bp | CGCCACGCATGCTCACCCTCTGGGGCAATCGGGGGG |
| MT_substrate strand_9bp | GGCAGAGATGGCG |
| MT_enzyme strand_9bp | CGCCACGCATGCTCACCCTCTGGGGCAATCGGGGGG |
| MT_substrate strand_10bp | GGCAGAGATGGCGC |
| MT_enzyme strand_10bp | GCGCCACGCATGCTCACCCTCTGGGGCAATCGGGGGG |
| MT_substrate strand_11bp | GGCAGAGATGGCGCT |
| MT_enzyme strand_11bp | AGTGCCACGCATGCTCACCCTCTGGGGCAATCGGGGGG |
| MT_substrate strand_12bp | GGCAGAGATGGCGCTG |
| MT_enzyme strand_12bp | CAGTGCCACGCATGCTCACCCTCTGGGGCAATCGGGGGG |
| MT_substrate strand_13bp | GGCAGAGATGGCGCTGT |
| MT_enzyme strand_13bp | ACAGTGCCACGCATGCTCACCCTCTGGGGCAATCGGGGGG |
| MT_substrate strand_14bp | GGCAGAGATGGCGCTGTC |
| MT_enzyme strand_14bp | GACAGTGCCACGCATGCTCACCCTCTGGGGCAATCGGGGGG |
| MT_substrate strand_15bp | GGCAGAGATGGCGCTGTCA |
| MT_enzyme strand_15bp | TGACAGTGCCACGCATGCTCACCCTCTGGGGCAATCGGGGGG |

**SUPPLEMENTARY FIGURES**


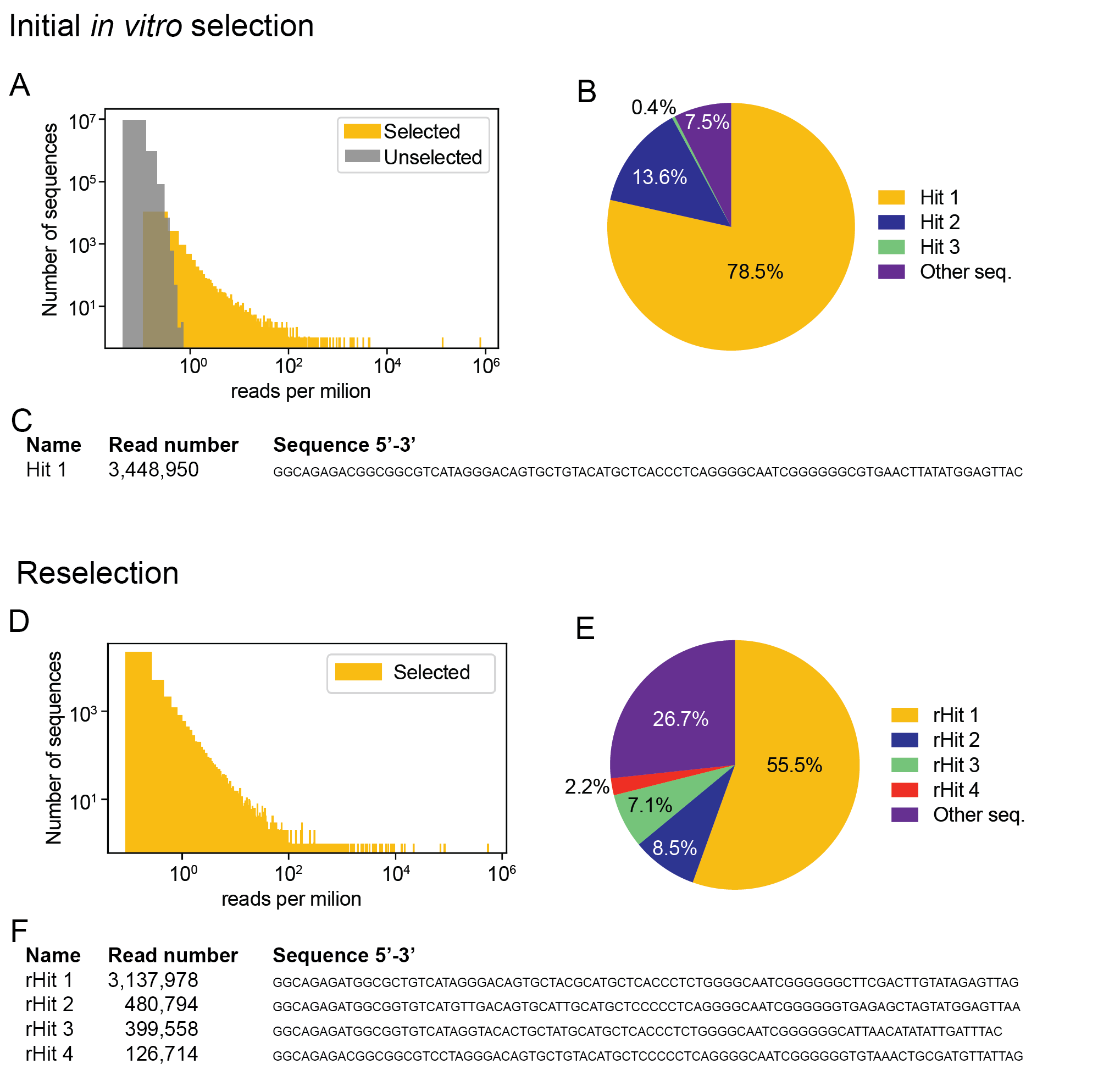
Supplementary Figure 1. Statistics from high-throughput sequencing datasets generated in selection experiments. (A) Distribution of read numbers (expressed in parts per million) in the initial library (Pool1 in Supplementary Table 1) before and after selection. (B) Chart showing the percent of total reads of the three most abundant sequences obtained in the selection. (C) Name, read number, and sequence of the most abundant deoxyribozyme obtained in the initial selection. (D) Distribution of read numbers (expressed in parts per million) in the reselection library (Pool2 in Supplementary Table 1) after selection. (E) Chart showing the percent of total reads of the four most abundant sequences obtained in the reselection. (F) Name, read number, and sequence of the four most abundant deoxyribozymes obtained in the reselection.


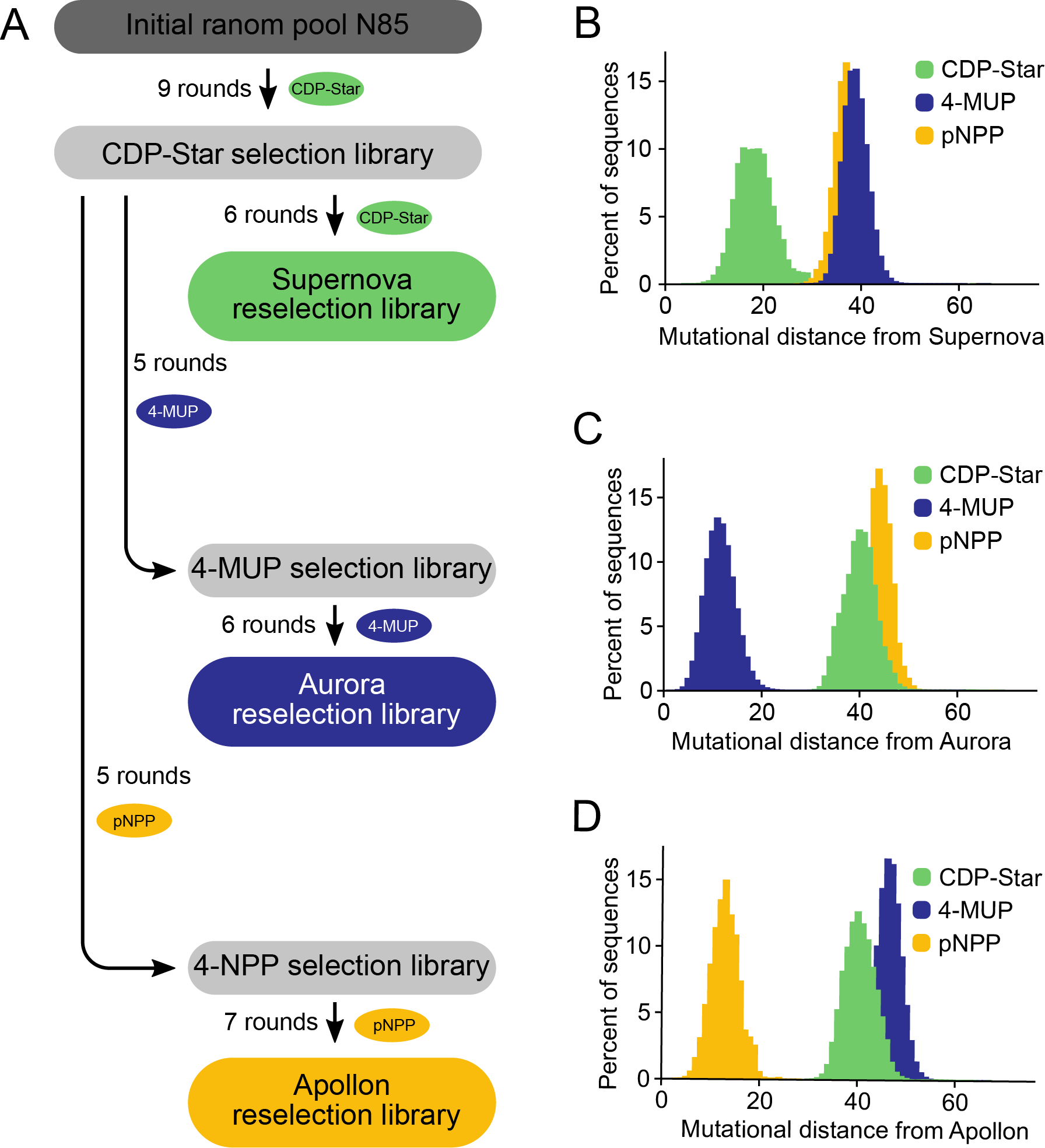
Supplementary Figure 2. Selections using different substrates yielded deoxyribozymes that are also distinct. (A) Workflow of selections in which deoxyribozymes that react with CDP-Star (10), 4-MUP (12), or pNPP (this study) were isolated. (B) Distribution of mutational distances of deoxyribozymes isolated in selections using these substrates relative to Supernova (which was identified by selecting for deoxyribozymes that react with CDP-Star). (C) Distribution of mutational distances of deoxyribozymes isolated using these substrates relative to Aurora (which was identified by selecting for deoxyribozymes that react with 4-MUP). (D) Distribution of mutational distances of deoxyribozymes isolated using these substrates relative to Apollon (which was identified by selecting for deoxyribozymes that react with pNPP).


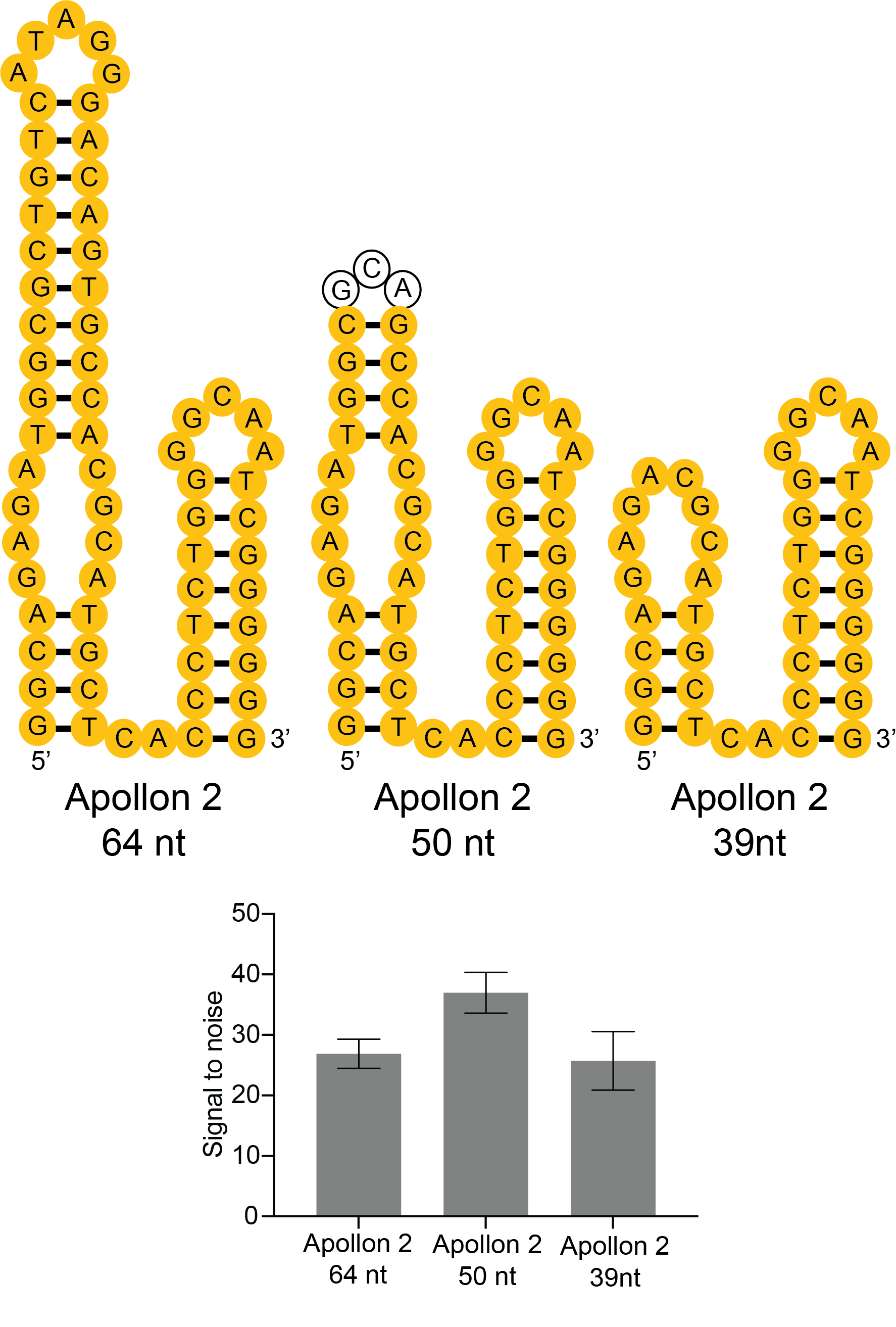
Supplementary Figure 3. Identification of the minimized catalytic core of Apollon. Above: deletion mutants of Apollon. In the case of the Apollon 2 64-nt variant, 21 nucleotides were deleted from the 3' end of the full-length deoxyribozyme. In the case of the Apollon 2 50-nt variant, 21 nucleotides were deleted from the 3' end of the full-length deoxyribozyme and nucleotides 13-29 were replaced with a GCA loop. And in the case of the Apollon 2 39-nt variant, 21 nucleotides were deleted from the 3' end of the full-length deoxyribozyme, and Stem 2 was also removed. Below: signal to noise ratios of the mutants. Reactions contained 30 μM DNA and 100 μM pNPP. Buffer contained 200 mM KCl, 1mM ZnCl_2_, and 50 mM HEPES pH 7.4. Absorbance at 405 nm was measured after 4 hours using a TECAN Infinite M200 Pro plate reader. Signal to noise ratio is defined as the absorbance at 405 nm in the presence of deoxyribozyme divided by the absorbance at 405 nm in the absence of deoxyribozyme. Columns show average values from three experiments, and error bars represent one standard deviation.


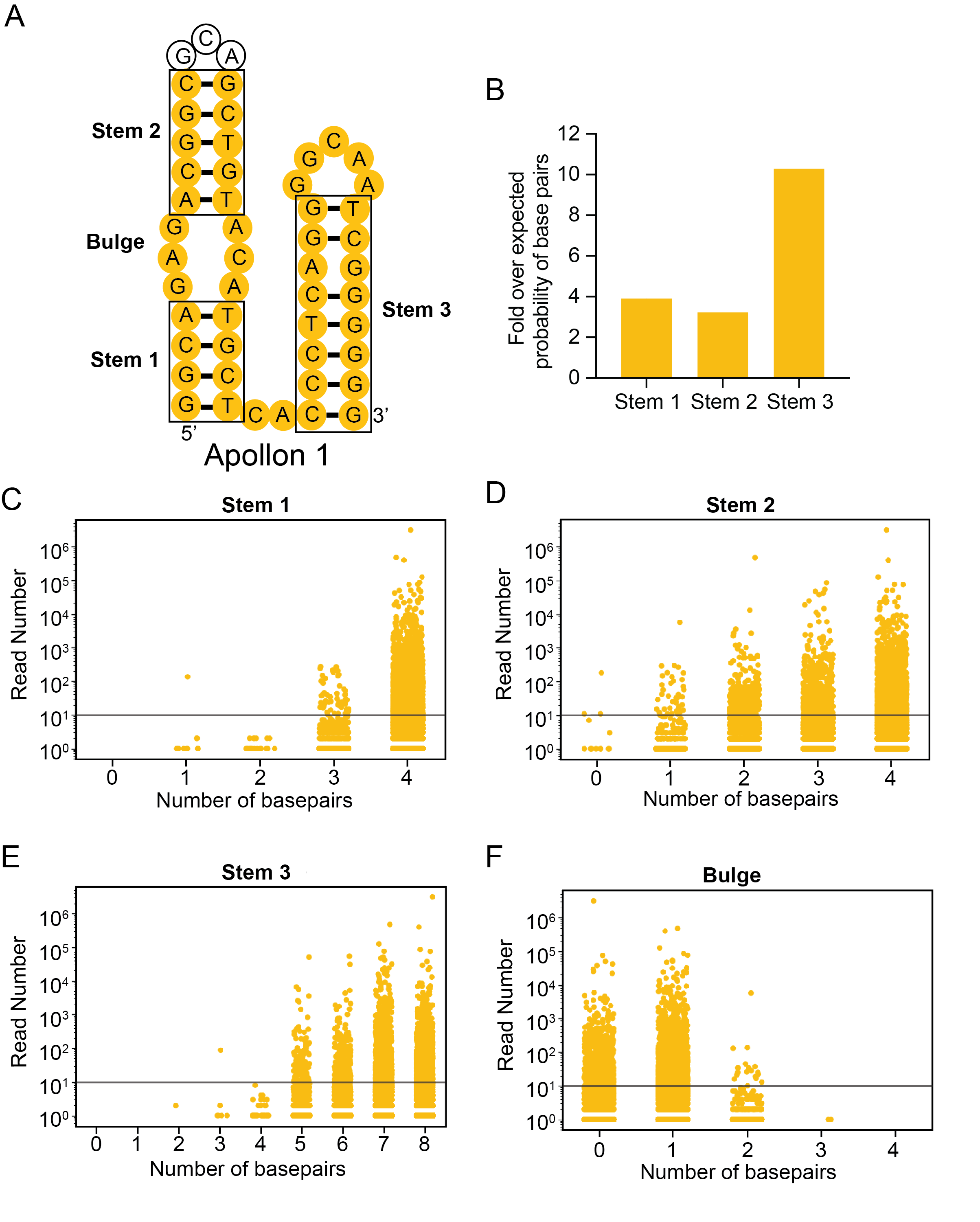
Supplementary Figure 4. Secondary structure of Apollon. (A) Secondary structure model of Apollon. Base pairs are shown using solid black lines. (B) Graph showing the frequency of variants in the evolved library with all base pairs in Stem 1, Stem 2, or Stem 3 divided by their frequency in the starting library. (C) Extent of pairing in Stem 1 as a function of read number in the evolved library. (D) Extent of pairing in Stem 2 as a function of read number in the evolved library. (E) Extent of pairing in Stem 3 as a function of read number in the evolved library. (F) Extent of pairing in the bulge between Stem 1 and Stem 2 as a function of read number in the evolved library.


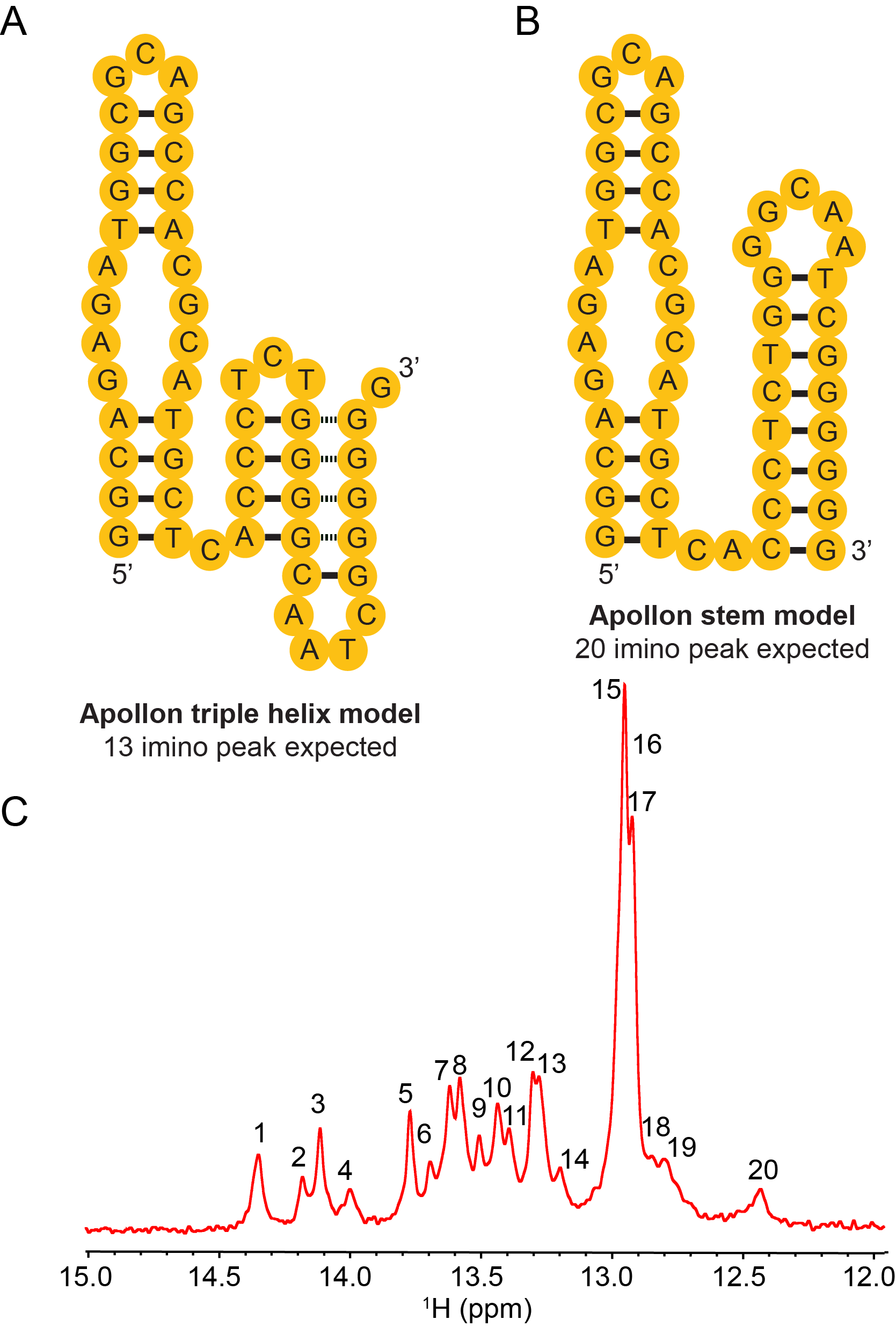
Supplementary Figure 5. NMR suggests that Stem 3 in Apollon forms a canonical helix rather than a triple helix. (A) Possible secondary structure model of Apollon that contains a purine-motif triple helix like that in the Supernova deoxyribozyme (reference 10 of the main text). (B) Secondary structure model for Apollon proposed in this study. (C) Proton NMR spectrum of Apollon. The number of signals is consistent with the model proposed in panel B but not that shown in panel A.


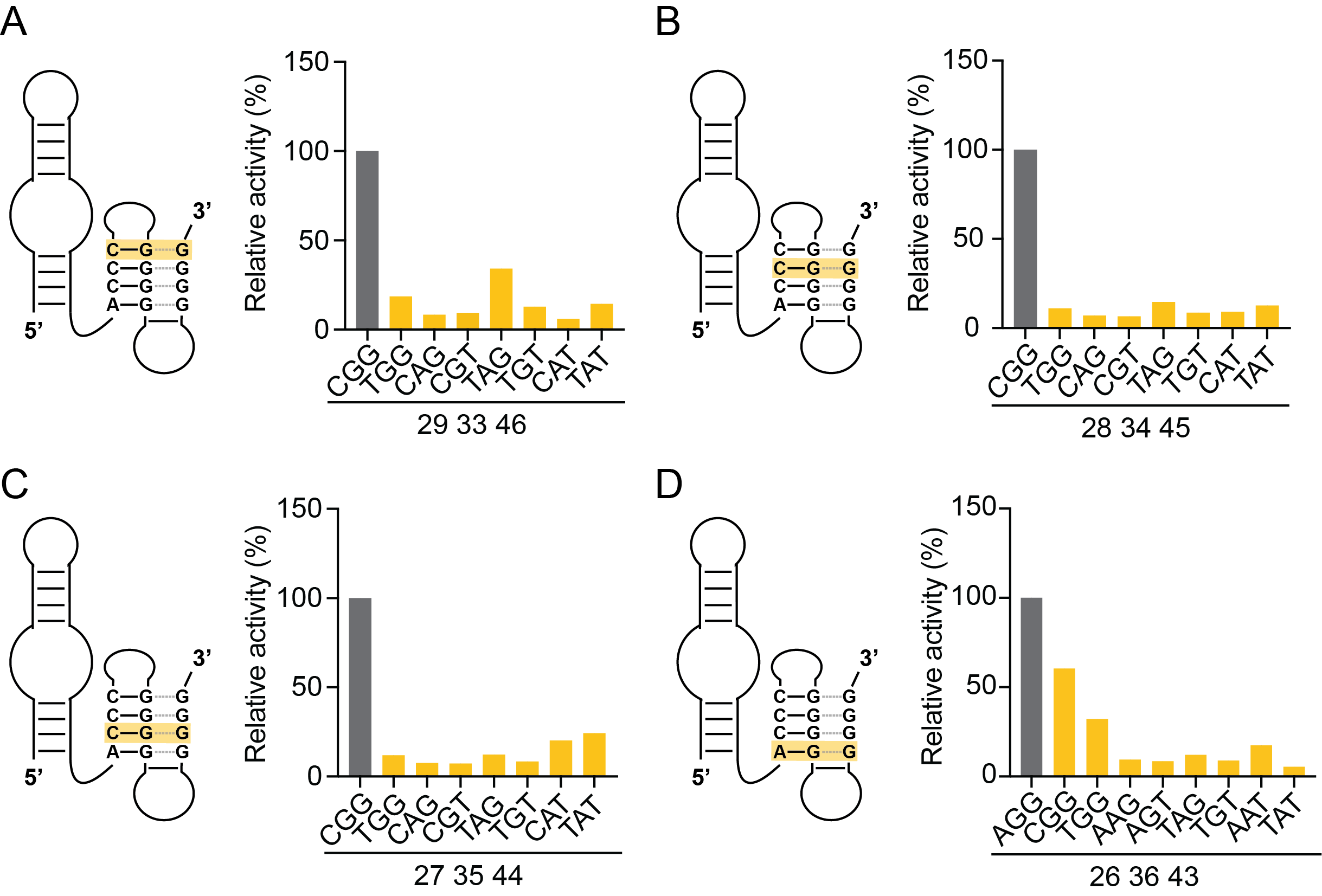
Supplementary Figure 6. Mutagenesis experiments do not support the hypothesis that Stem 3 of Apollon contains a purine-motif triple helix. (A) Left: secondary structure model with the putative 29-33-46 base triple highlighted in yellow. Right: triple mutant cycle in which the putative CGG triple is converted to a TAT triple. Activity is normalized to that of Apollon 2. (B) Same, but for the 28-34-45 base triple. (C) Same, but for the 27-35-44 base triple. (D) Same, but for the 26-36-43 triple. Reactions contained 30 μM DNA and 100 μM pNPP. The buffer contained 200 mM KCl, 1mM ZnCl_2_, and 50 mM HEPES pH 7.4. Absorbance at 405 nm was measured after 4 hours using a TECAN Infinite M200 Pro plate reader.


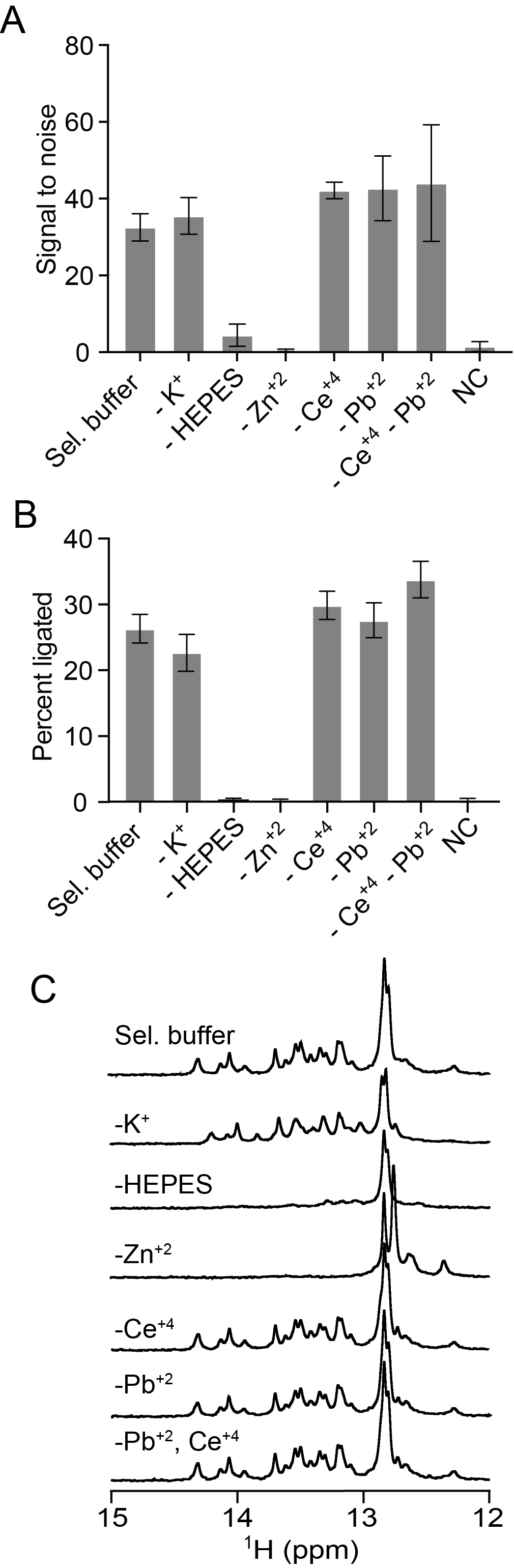
Supplementary Figure 7. Apollon requires HEPES and zinc for activity and folding. (A) Signal to noise ratios of Apollon in a series of buffers in which different components were omitted. The original selection buffer (labeled "Sel. buffer") contained 200 mM KCl, 1 μM CeO_2_, 0.1 μM PbCl_2_, 1 mM ZnCl_2_, and 50 mM HEPES pH 7.4. Reactions contained 30 μM Apollon and 100 μM pNPP. After incubating for 4 hours, the absorbance at 405 nm was measured using a TECAN Infinite M200 Pro plate reader. Signal to noise ratio is defined as the absorbance at 405 nm in the presence of deoxyribozyme divided by the absorbance at 405 nm in the absence of deoxyribozyme. (B) Same as in panel A, but showing the percent of ligated Apollon measured using the ligation assay. The percent ligated was determined after incubating 1 μM Apollon with 1 mM pNPP for 1 hour. (C) Proton NMR spectra of Apollon in the same buffers used in panel A. Spectra were measured using 300 μM Apollon and 450 μM pNPP. Columns in panels A and B show the values from three experiments, and error bars represent one standard deviation. Experiments were performed using Apollon 2.


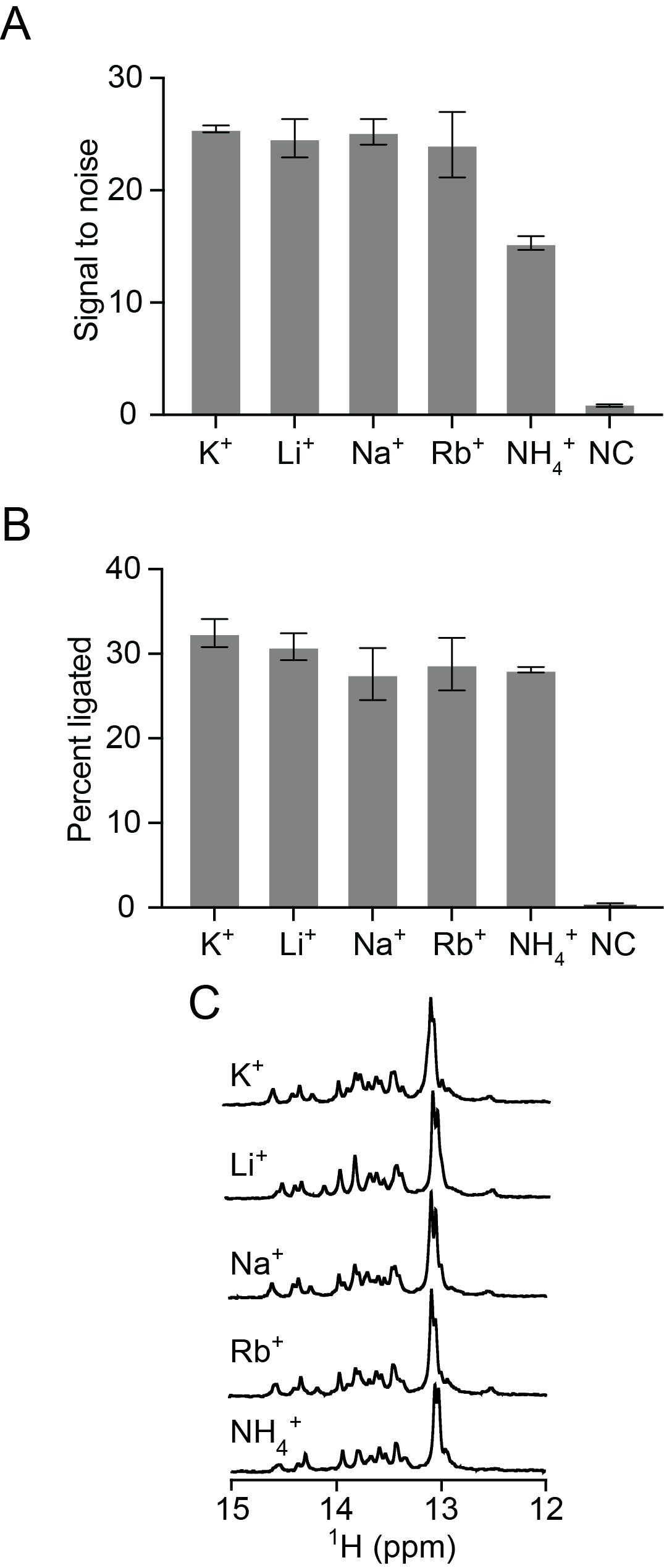
Supplementary Figure 8. Apollon is active in a wide range of monovalent metal ions. (A) Signal to noise ratios of Apollon in a series of buffers in which potassium was replaced with other monovalent metal ions. Buffers contained the indicated monovalent cation at a concentration of 200 mM, 1 mM ZnCl_2_, and 50 mM HEPES pH 7.4. Reactions contained 30 μM Apollon and 100 μM pNPP. After incubating for 4 hours, absorbance at 405 nm was measured using a TECAN Infinite M200 Pro plate reader. The signal to noise ratio is defined as the absorbance at 405 nm in the presence of deoxyribozyme divided by the absorbance at 405 nm in the absence of deoxyribozyme. (B) Same as in panel A, but showing the percent of ligated Apollon measured using the ligation assay. The percent ligated was determined after incubating 1 μM Apollon with 1 mM pNPP for 1 hour. (C) Proton NMR spectra of Apollon in the same buffer used in panel A. Spectra were measured using 300 μM Apollon and 450 μM pNPP. Columns in panels A and B show the values from three experiments, and error bars represent one standard deviation. Experiments were performed using Apollon 2.


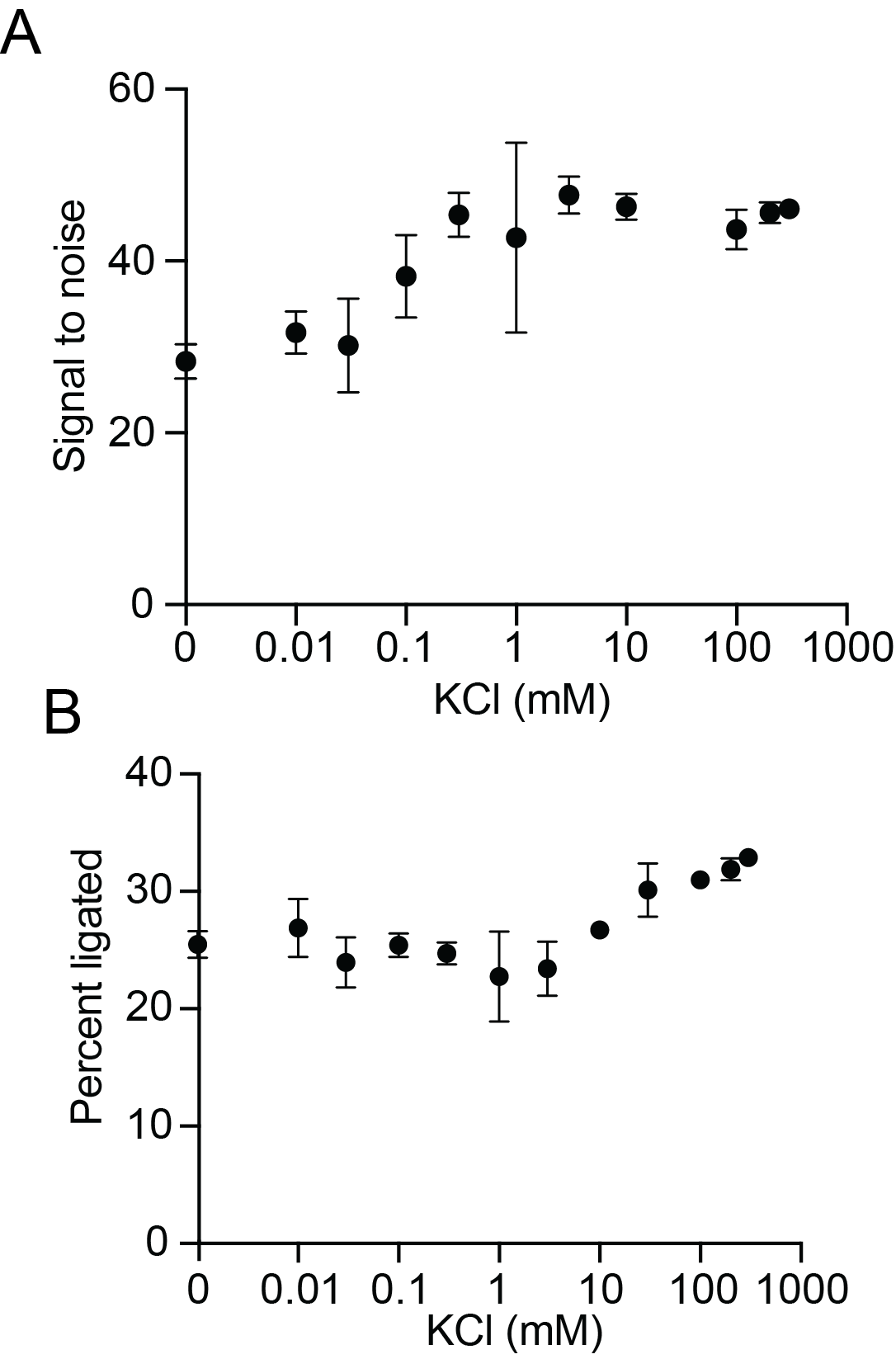
Supplementary Figure 9. Apollon activity is not dependent on KCl concentration. (A) Signal to noise ratio of Apollon over a range of potassium concentrations. Buffers contained the indicated concentration of KCl, 1 mM ZnCl_2_, and 50 mM HEPES pH 7.4. Reactions contained 30 μM Apollon and 100 μM pNPP. After incubating for 4 hours, absorbance at 405 nm was measured using a TECAN Infinite M200 Pro plate reader. Signal to noise ratio is defined as the absorbance at 405 nm in the presence of deoxyribozyme divided by the absorbance at 405 nm in the absence of deoxyribozyme. (B) Same experiment as in A, but showing the percent of ligated Apollon measured using the ligation assay. The percent ligated was determined after incubating 1 μM Apollon with 1 mM pNPP for 1 hour. Points show the values from three experiments, and error bars represent one standard deviation. Experiments were performed using Apollon 2.


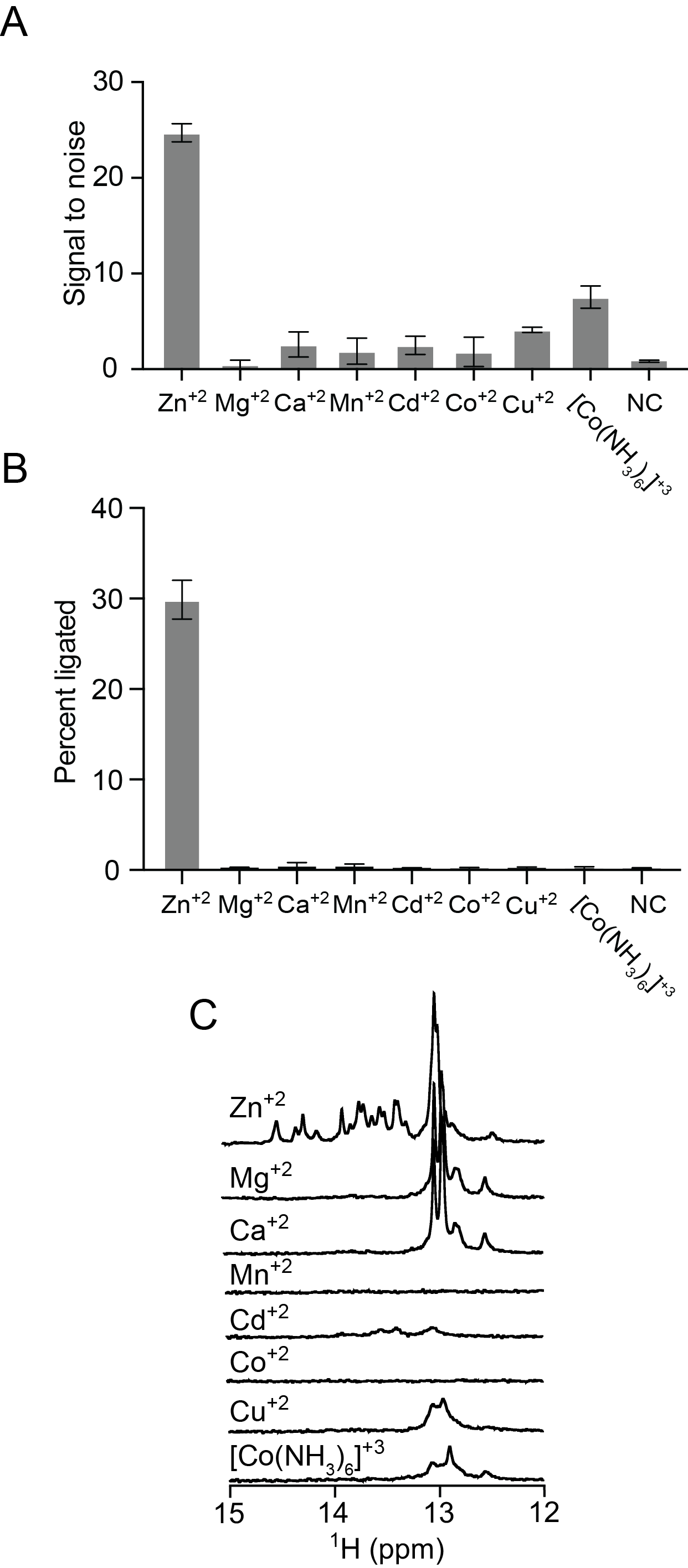
Supplementary Figure 10. Apollon is active in the presence of zinc, but not in the presence of other divalent metal ions. (A) Signal to noise ratio of Apollon in a series of buffers in which zinc was replaced with other divalent metal ions. Buffers contained the indicated divalent cation at a concentration of 1 mM, 200 mM KCl, and 50 mM HEPES pH 7.4. Reactions contained 30 μM Apollon and 100 μM pNPP. After incubating for 4 hours, absorbance at 405 nm was measured using a TECAN Infinite M200 Pro plate reader. Signal to noise ratio is defined as the absorbance at 405 nm in the presence of deoxyribozyme divided by the absorbance at 405 nm in the absence of deoxyribozyme. (B) Same as in panel A, but showing the percent of ligated Apollon measured using the ligation assay. The percent ligated was determined after incubating 1 μM Apollon with 1 mM pNPP for 1 hour. (C) Proton NMR spectra of Apollon in the same buffer used in panel A. Spectra were measured using 300 μM Apollon and 450 μM pNPP. Columns in panels A and B show average values from three experiments, and error bars represent one standard deviation. Experiments were performed using Apollon 2.


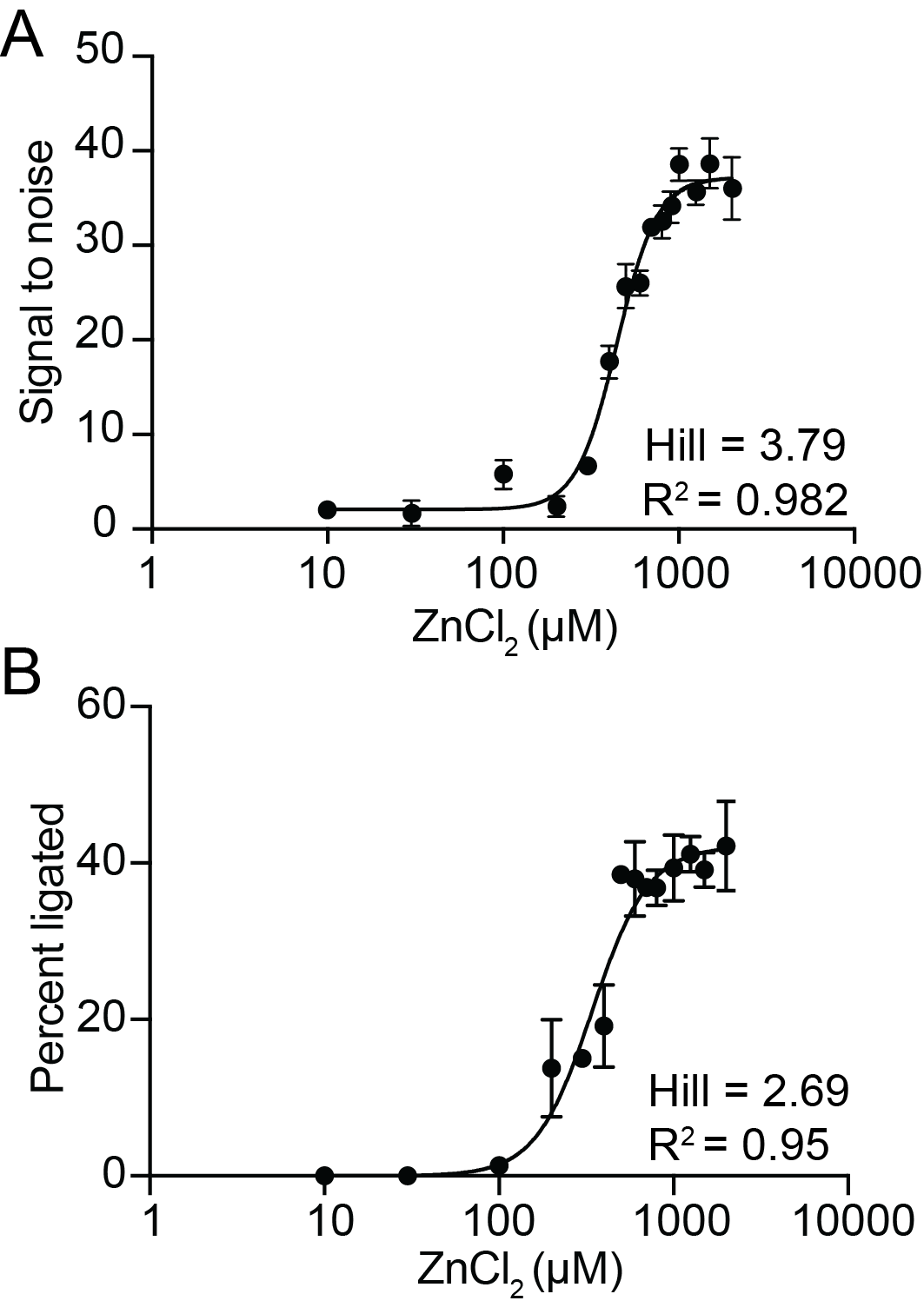
Supplementary Figure 11. Cooperative effect of zinc on Apollon catalytic activity. (A) Signal to noise ratio of Apollon as a function of zinc concentration. Buffers contained the indicated concentration of ZnCl_2_, 200 mM KCl, and 50 mM HEPES pH 7.4. Reactions contained 30 μM Apollon and 100 μM pNPP. After incubating for 4 hours, absorbance at 405 nm was measured using a TECAN Infinite M200 Pro plate reader. Signal to noise ratio is defined as the absorbance at 405 nm in the presence of deoxyribozyme divided by the absorbance at 405 nm in the absence of deoxyribozyme. (B) Same experiment as in panel A, but showing the percent of phosphorylated Apollon measured using the ligation assay. The percent ligated was determined after incubating 1 μM Apollon with 1 mM pNPP for 1 hour. Experiments were performed using Apollon 2. Points show average values from three experiments, and error bars represent one standard deviation. Data were fitted with Hill equation (3) using Prims10 software.


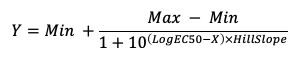
 (3)

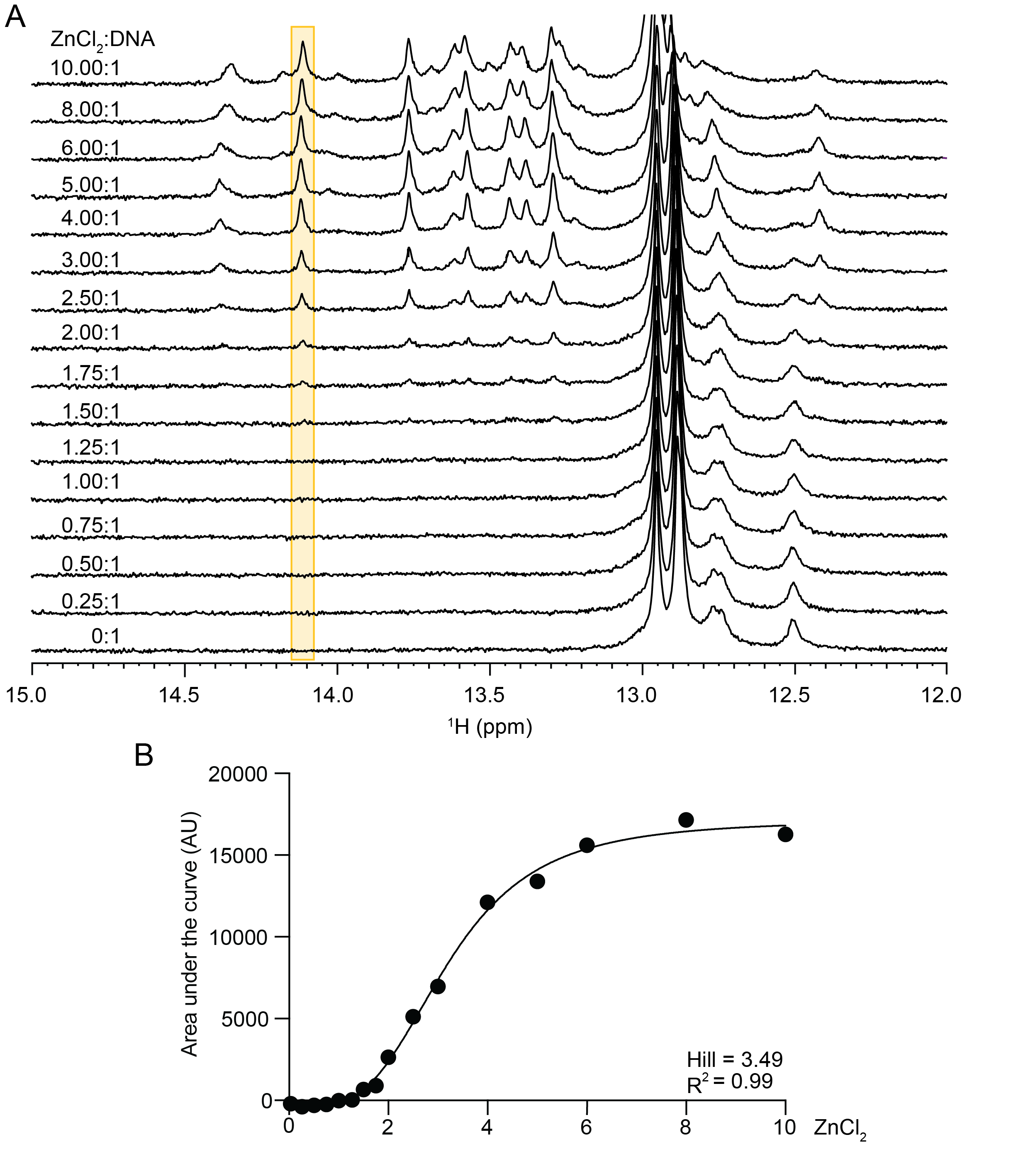
Supplementary Figure 12. Cooperative effect of zinc on Apollon folding. (A) Proton NMR spectra of Apollon over a series of concentrations of ZnCl_2_. Spectra were measured in the presence of 300 μM DNA, 450 μM pNPP, 200 mM KCl, and 50 mM HEPES pH 7.4. (B) Graph showing the area under the curve of the second peak from the left in panel A (indicated with a yellow box). Experiments were performed using Apollon 2. Data were fitted with Hill equation (3) using Prims10 software.


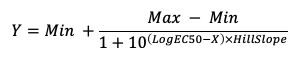
 (3)

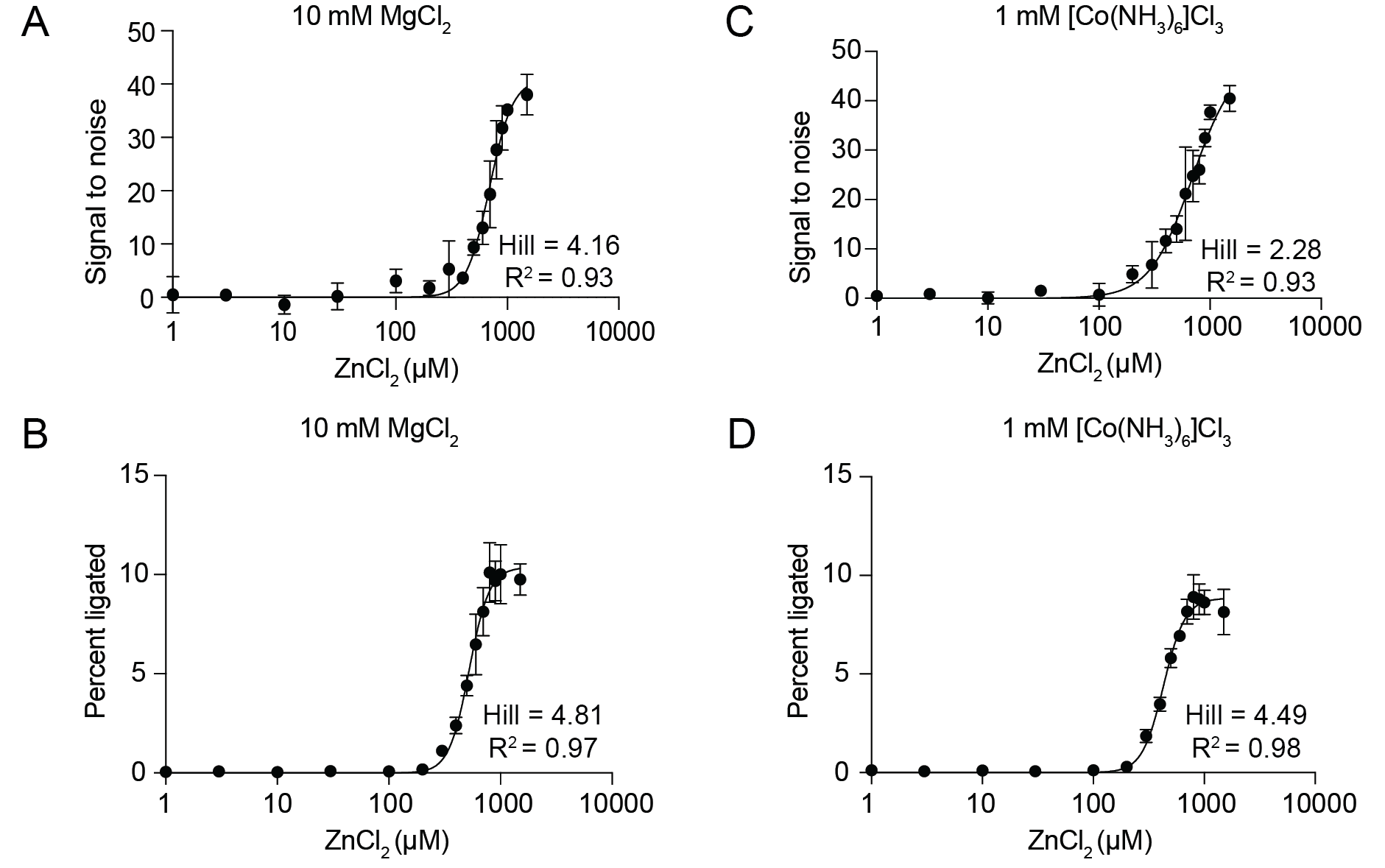
Supplementary Figure 13. Cooperative effect of zinc on Apollon catalytic activity in the presence of other metal ions. (A) Signal to noise ratio of Apollon as a function of zinc concentration in the presence of 10 mM MgCl_2_. Reactions contained 30 μM Apollon, 100 μM pNPP, the indicated concentration of ZnCl_2_, 200 mM KCl, 50 mM HEPES pH 7.4, and 10 mM MgCl_2_. After incubating for 4 hours, absorbance at 405 nm was measured using a TECAN Infinite M200 Pro plate reader. Signal to noise ratio is defined as the absorbance at 405 nm in the presence of deoxyribozyme divided by the absorbance at 405 nm in the absence of deoxyribozyme. (B) Same experiment as in panel A, but showing the percent of phosphorylated Apollon measured using the ligation assay. The percent ligated was determined after incubating 1 μM Apollon with 1 mM pNPP for 1 hour. Points show average values from three experiments, and error bars represent one standard deviation. (C) Same experiment as in panel A, but in the presence of 1 mM [Co(NH_3_)_6_]Cl_3_ rather than 10 mM MgCl_2_. (D) Same experiment as in panel B, but in the presence of 1 mM [Co(NH_3_)_6_]Cl_3_ rather than 10 mM MgCl_2_.


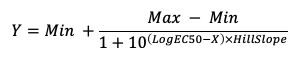
 (3)

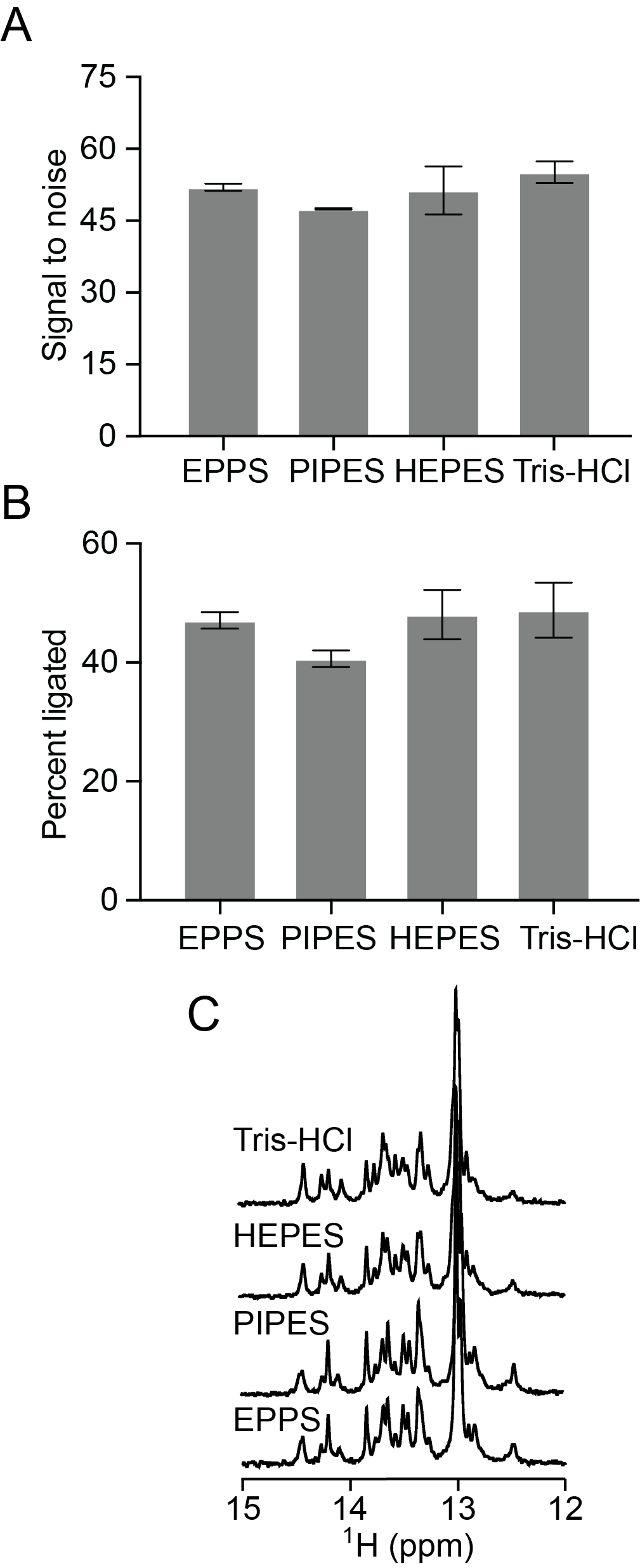
Supplementary Figure 14. Apollon is active in a wide range of buffering agents. (A) Signal to noise ratio of Apollon in a series of buffers in which HEPES was replaced with another buffering agent. Reactions contained the indicated buffering agent at a concentration of 50 mM at pH 7.4 as well as 200 mM KCl and 1 mM ZnCl_2_. Reactions contained 30 μM Apollon and 100 μM pNPP. After incubating for 4 hours, absorbance at 405 nm was measured using a TECAN Infinite M200 Pro plate reader. Signal to noise ratio is defined as the absorbance at 405 nm in the presence of deoxyribozyme divided by the absorbance at 405 nm in the absence of deoxyribozyme. (B) Same as in panel A, but showing the percent of ligated Apollon measured using the ligation assay. The percent ligated was determined after incubating 1 μM Apollon with 1 mM pNPP for 1 hour. (C) Proton NMR spectra of Apollon in the same buffer used in panel A. Spectra were measured using 300 μM Apollon and 450 μM pNPP. Columns in panels A and B show average values from three experiments, and error bars represent one standard deviation. Experiments were performed using Apollon 2.


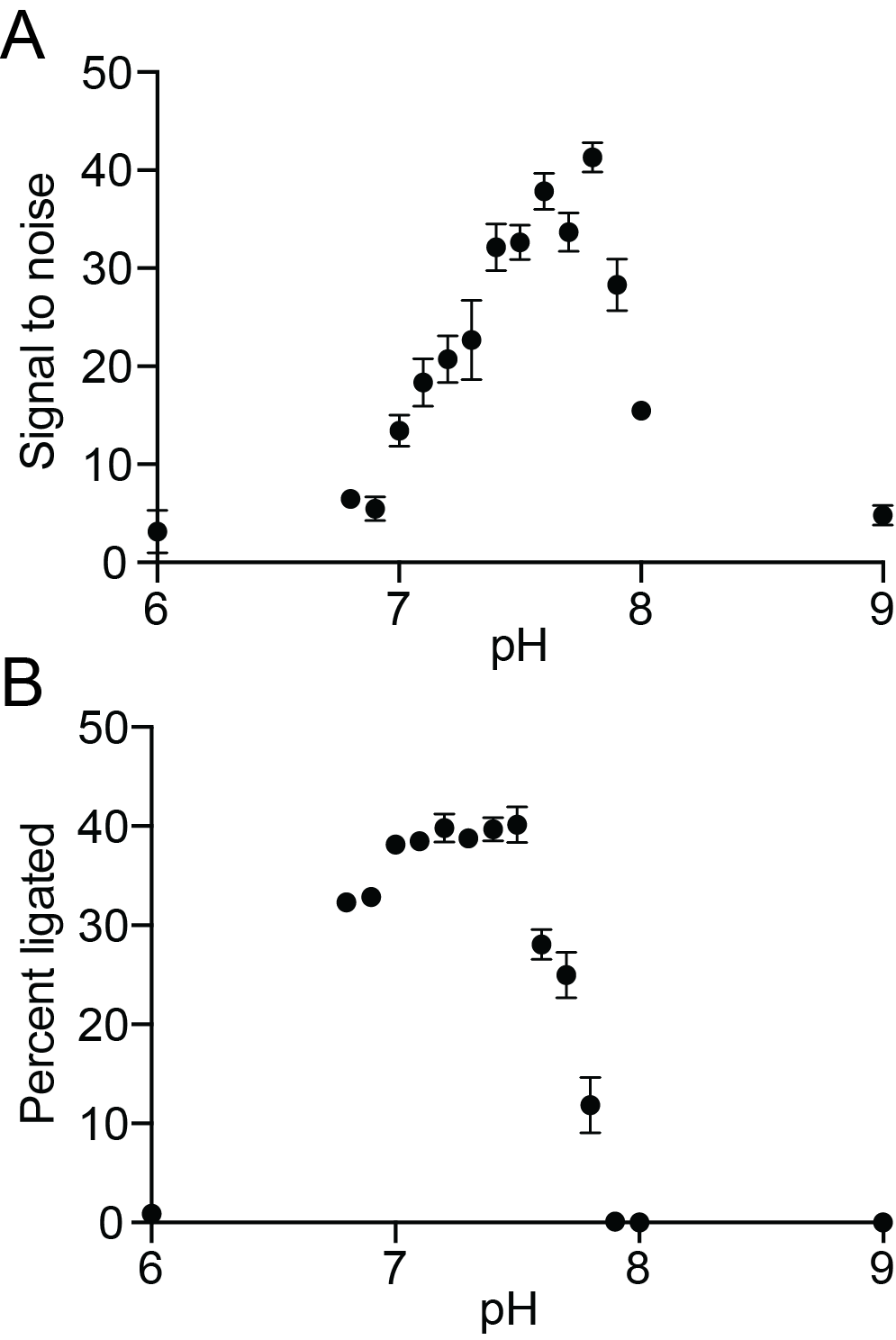
Supplementary Figure 15. Effect of pH on Apollon catalytic activity. (A) Signal to noise ratio of Apollon as a function of pH. Buffers contained 50 mM HEPES at the indicated pH, 200 mM KCl, and 1 mM ZnCl_2_. Reactions contained 30 μM Apollon and 100 μM pNPP. After incubating for 4 hours, absorbance at 405 nm was measured using a TECAN Infinite M200 Pro plate reader. Signal to noise ratio is defined as the absorbance at 405 nm in the presence of deoxyribozyme divided by the absorbance at 405 nm in the absence of deoxyribozyme. (B) Same as in panel A, but showing the percent of ligated Apollon measured using the ligation assay. The percent ligated was determined after incubation of 1 μM Apollon with 1 mM pNPP for 1 hour. Points show average values from three experiments, and error bars represent one standard deviation. Experiments were performed using Apollon 2.


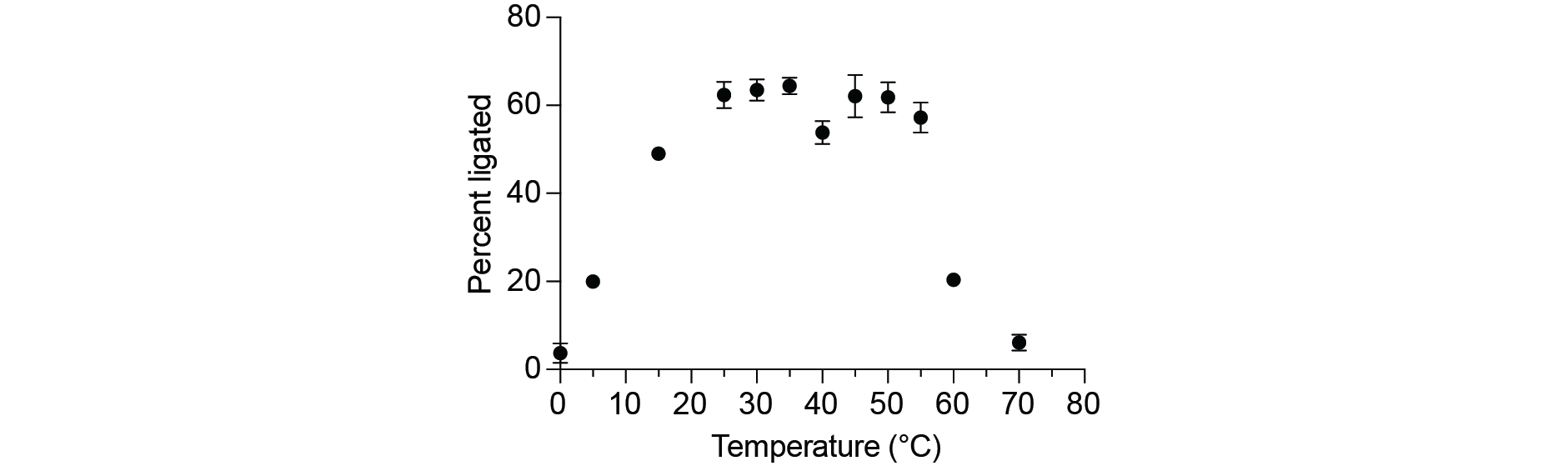
Supplementary Figure 16. Temperature dependence of Apollon as measured using the ligation assay. The pH of the buffering agent (HEPES) was adjusted as recommended by the manufacturer so that it would remain at pH 7.4 at different temperatures. Buffers contained 200 mM KCl, 1 mM ZnCl_2_ and 50 mM HEPES at the indicated pH. The percentage of ligated Apollon was determined after incubating 1 μM Apollon with 1 mM pNPP for 1 hour at the indicated temperature. Points show average values from three experiments, and error bars represent one standard deviation. Experiments were performed using Apollon 2.


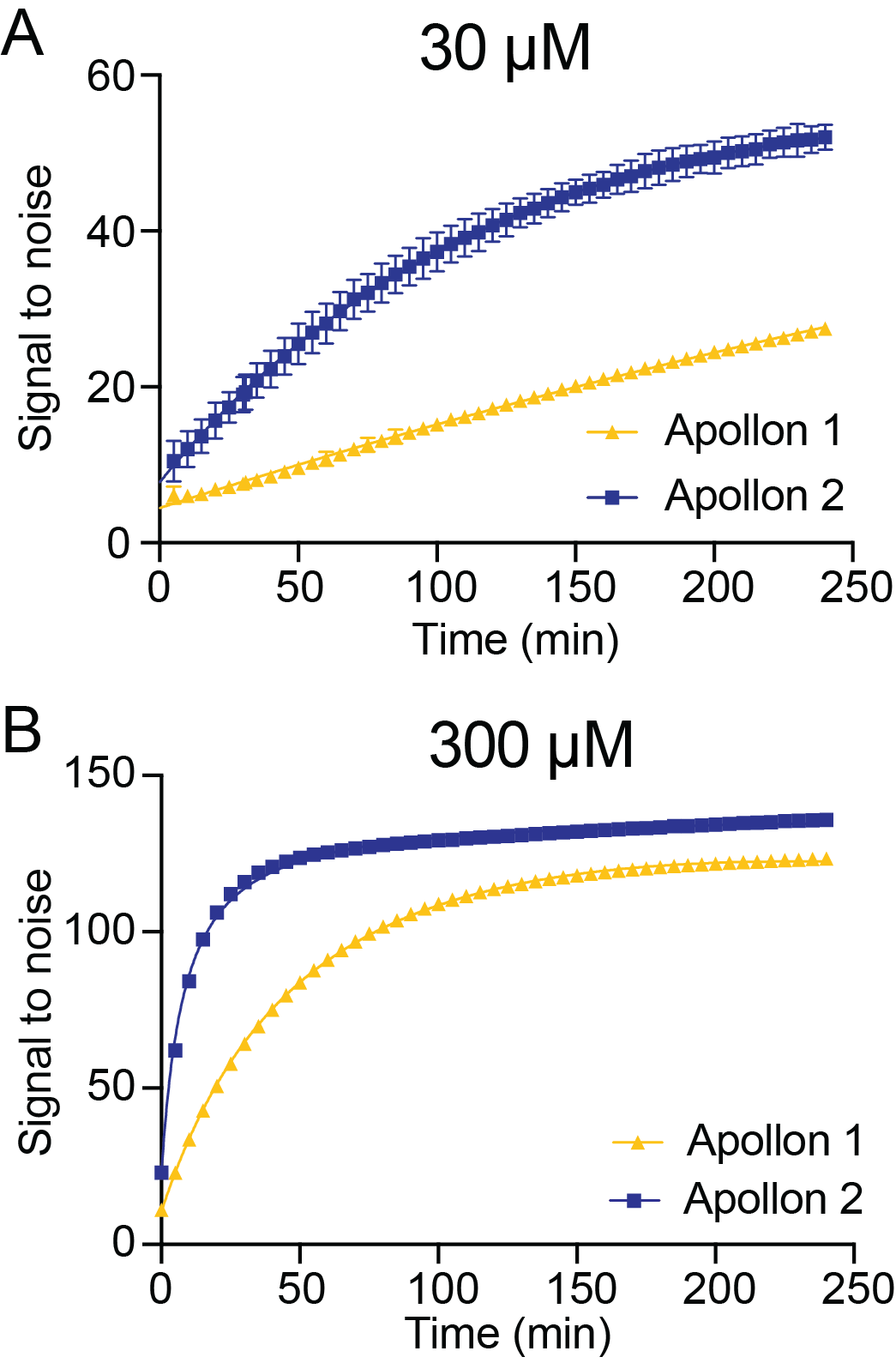
Supplementary Figure 17. Colorimetric reaction of Apollon 1 and Apollon 2. (A) Signal to noise ratio of color production of Apollon 1 and Apollon 2 at 30 μM deoxyribozyme concentration. (B) Same reaction as in panel A but at 300 μM deoxyribozyme concentration. Reactions contained 30 μM DNA and 100 μM pNPP or 300μM DNA and 1mM pNPP as well as 200mM KCl, 1mM ZnCl2, and 50mM HEPES pH 7.4. Absorbance at 405 nm was measured for 4 hours using a TECAN Infinite M200 Pro plate reader. Signal to noise ratio is defined as the absorbance at 405 nm in the presence of deoxyribozyme divided by the absorbance at 405 nm in the absence of deoxyribozyme. Points show average values from three experiments, and error bars represent one standard deviation. Experiments were performed using Apollon 1 and Apollon 2.


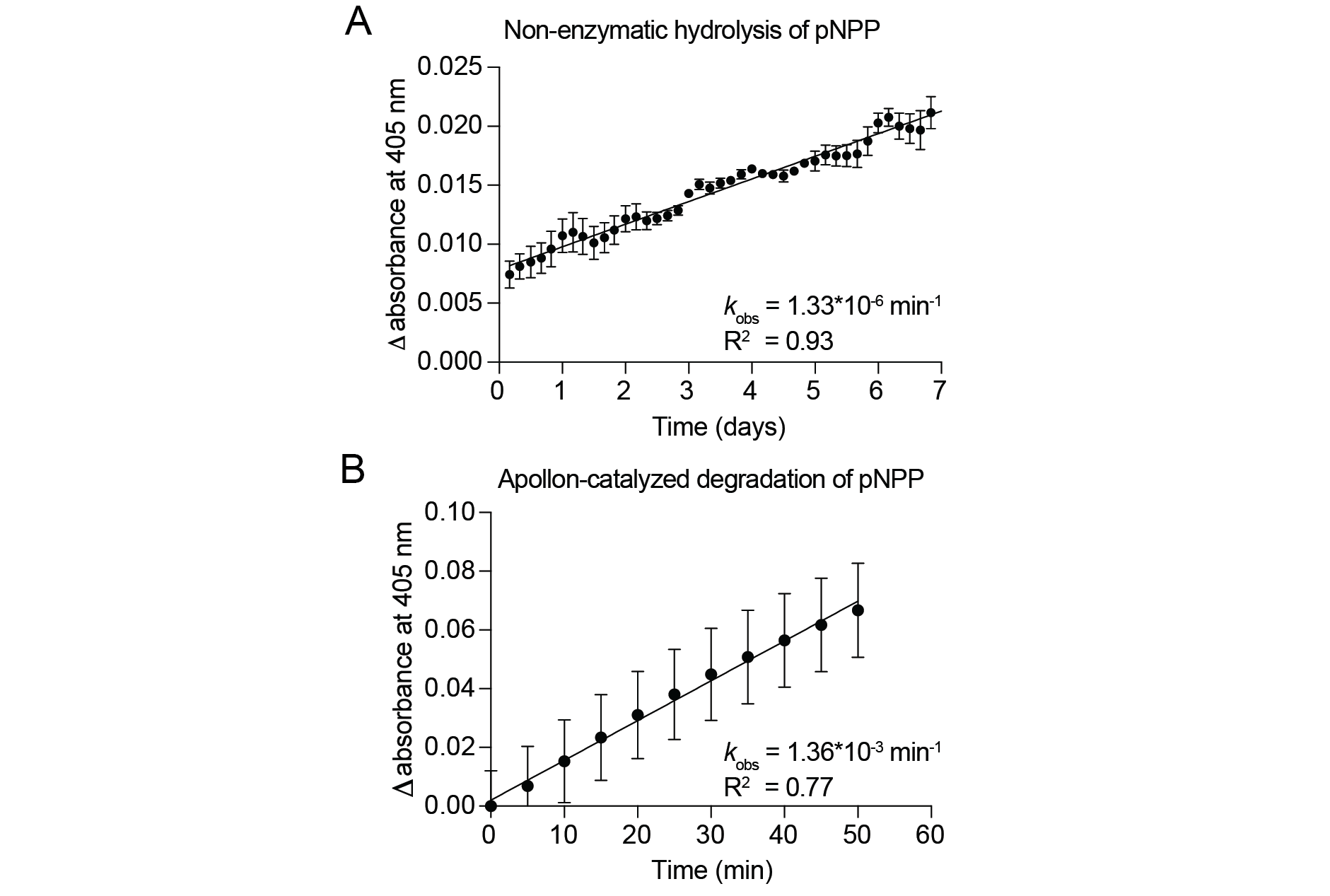
Supplementary Figure 18. Rate of nonenzymatic hydrolysis of pNPP. (A) Color produced by 1 mM pNPP over 7 days. (B) Color produced by 30 μM Apollon 2 in the presence of 100 μM pNPP over 1 hour. Both experiments were performed in a buffer containing 200 mM KCl, 1 mM ZnCl_2_, and 50 mM HEPES pH 7.4. Absorbance at 405 nm was measured using a TECAN Infinite M200 Pro plate reader. Points show average values from three experiments, and error bars represent one standard deviation. Data were fit by simple linear regression using Prims10 software.


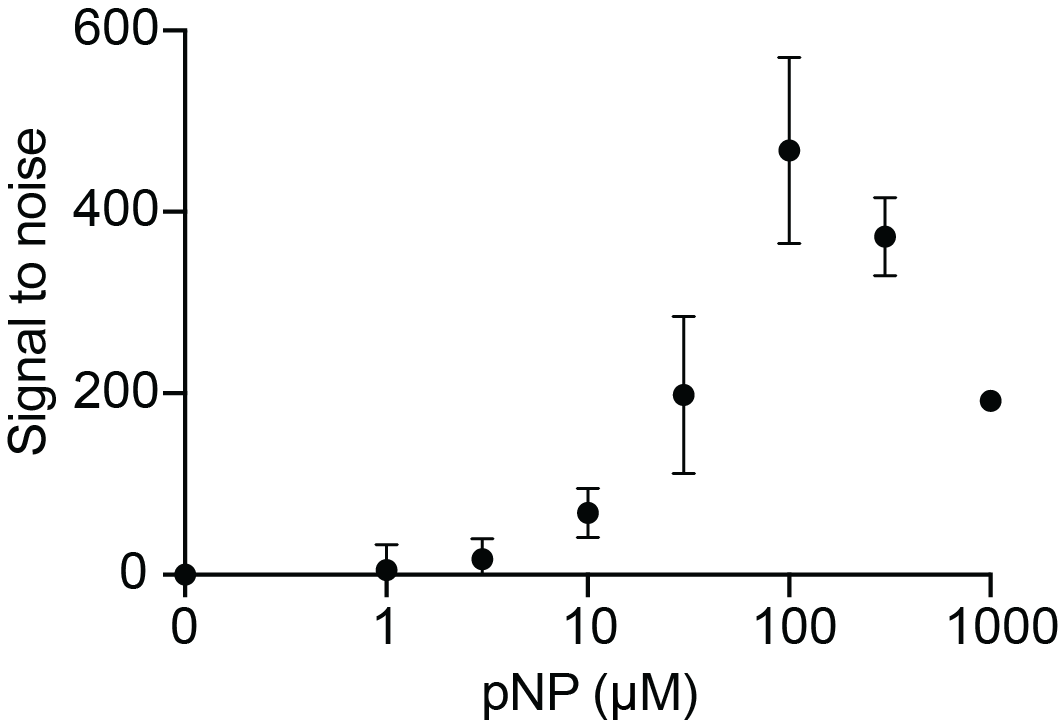
Supplementary Figure 19. Signal to noise ratio of a *para*-nitrophenol (pNP) synthetic standard. Solutions contained the indicated concentration of pNP as well as 200 mM KCl, 1 mM ZnCl_2_, and 50 mM HEPES pH 7.4. Absorbance at 405 nm was measured using a TECAN Infinite M200 Pro plate reader after incubating at room temperature for 4 hours. Signal to noise ratio is defined as the absorbance at 405 nm in the presence of the pNP divided by the absorbance at 405 nm in the presence of pNPP. Points show average values from three experiments, and error bars represent one standard deviation.


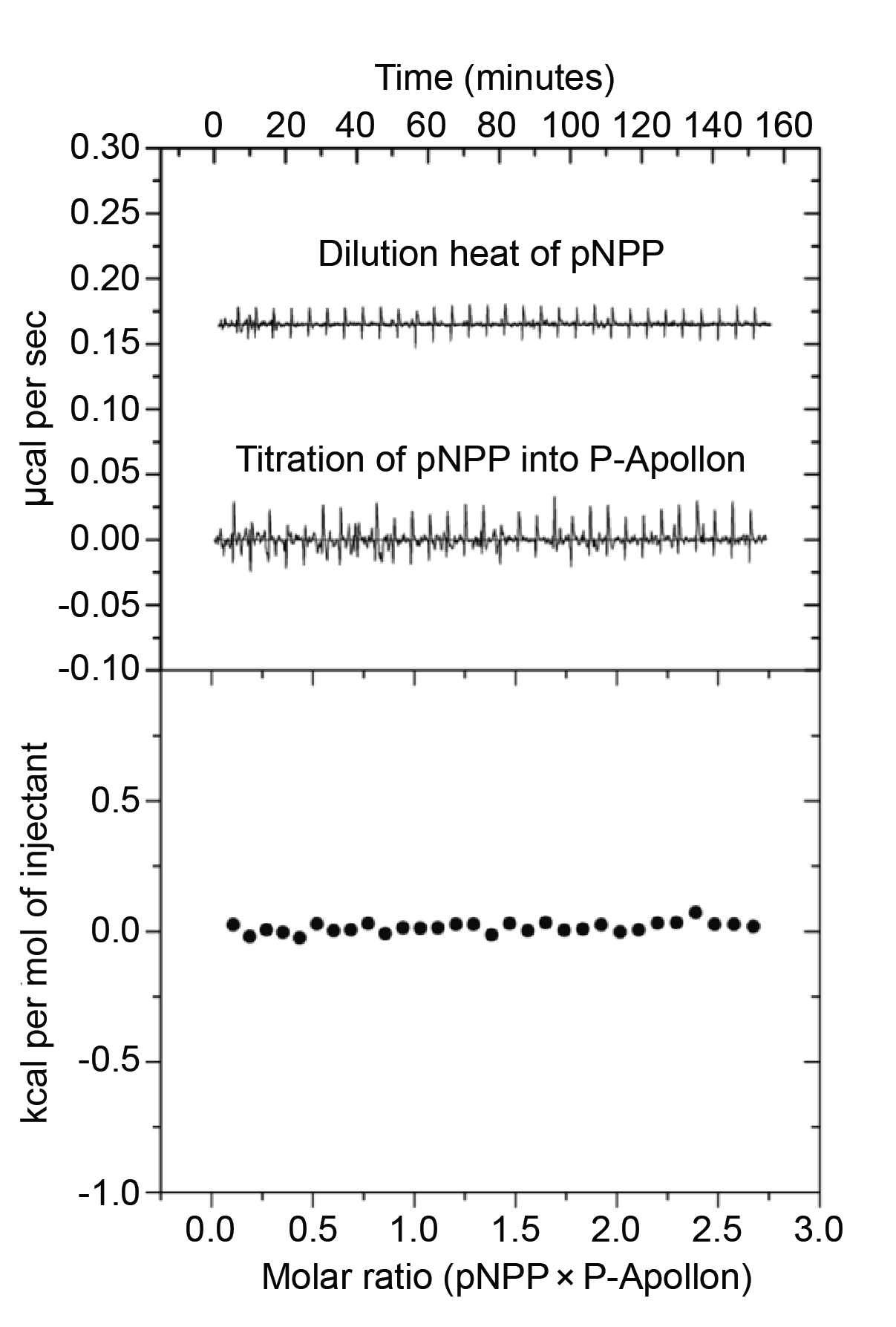
Supplementary Figure 20. 5' phosphorylated Apollon 2 does not bind pNPP. Isothermal calorimetric titration of 5' phosphorylated Apollon 2 with pNPP. Binding of pNPP to 5' phosphorylated Apollon 2 was measured using 101.3 μM DNA in 200 mM KCl, 50 mM HEPES pH 7.4, and 1 mM ZnCl_2_. Stepwise injections of typically 9 μl of 1.3 mM pNPP were performed until saturation was achieved.


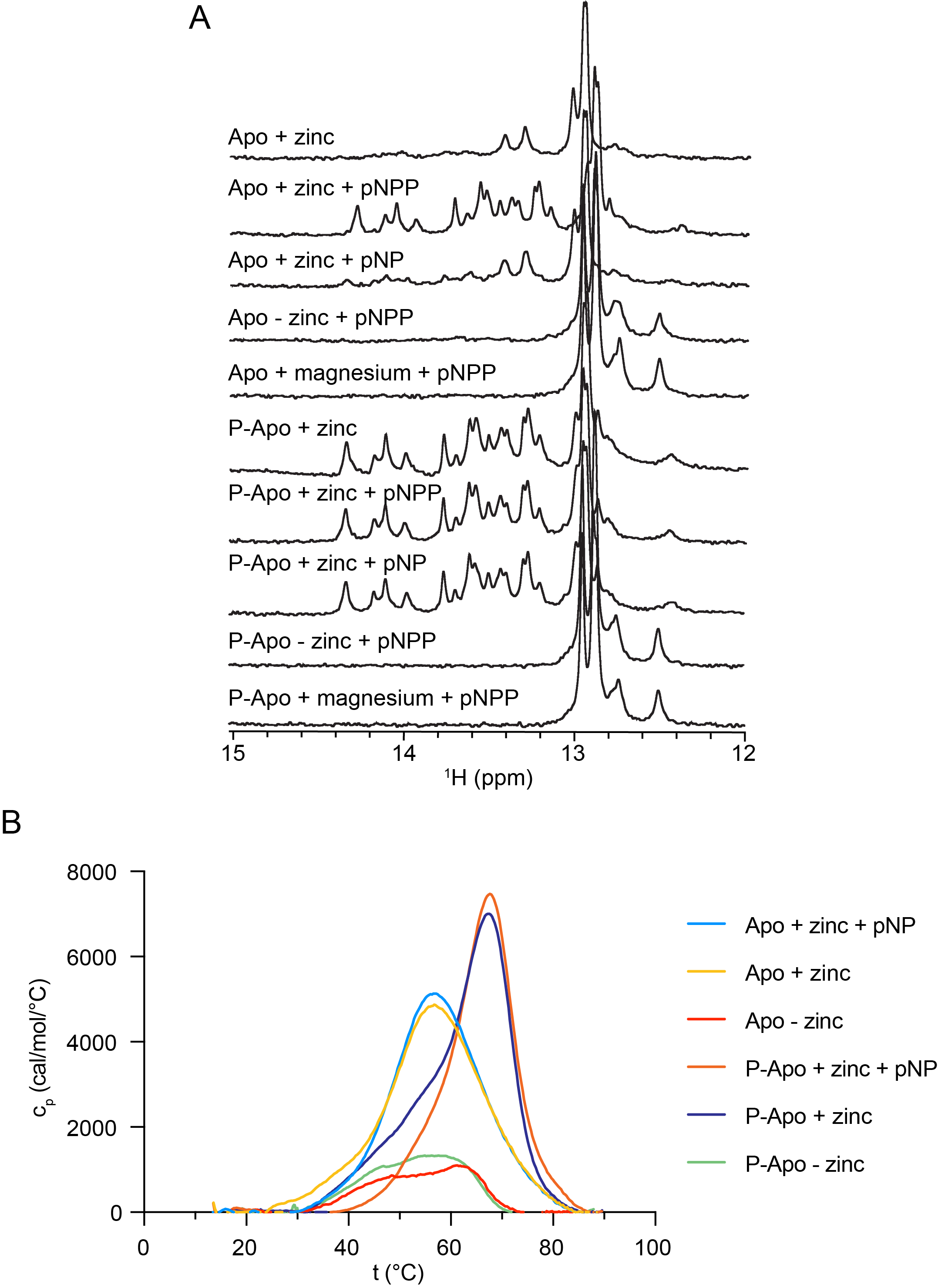
Supplementary Figure 21. Apollon requires zinc and a 5' phosphate to form a stable structure. (A) Proton NMR spectra of Apollon (which contains a 5' hydroxyl group and is labeled Apo) and P-Apollon (which contains a 5' phosphate group and is labeled P-Apo) with pNPP and zinc, with zinc but not pNPP, with pNP and zinc, with pNPP but not zinc, and with pNPP and magnesium instead of zinc. Spectra were measured in the presence of 300 μM DNA, 450 μM pNPP, 200 mM KCl, 50 mM HEPES pH 7.4, and 1 mM ZnCl_2_ if not stated otherwise. (B) Denaturation profiles of Apollon (labeled Apo) and P-Apollon (labeled P-Apo) with or without pNP and with or without zinc. Profiles were measured using 30 μM DNA in 200 mM KCl, 50 mM HEPES pH 7.4, 1 mM ZnCl_2_ and 60 μM pNP if not stated otherwise.


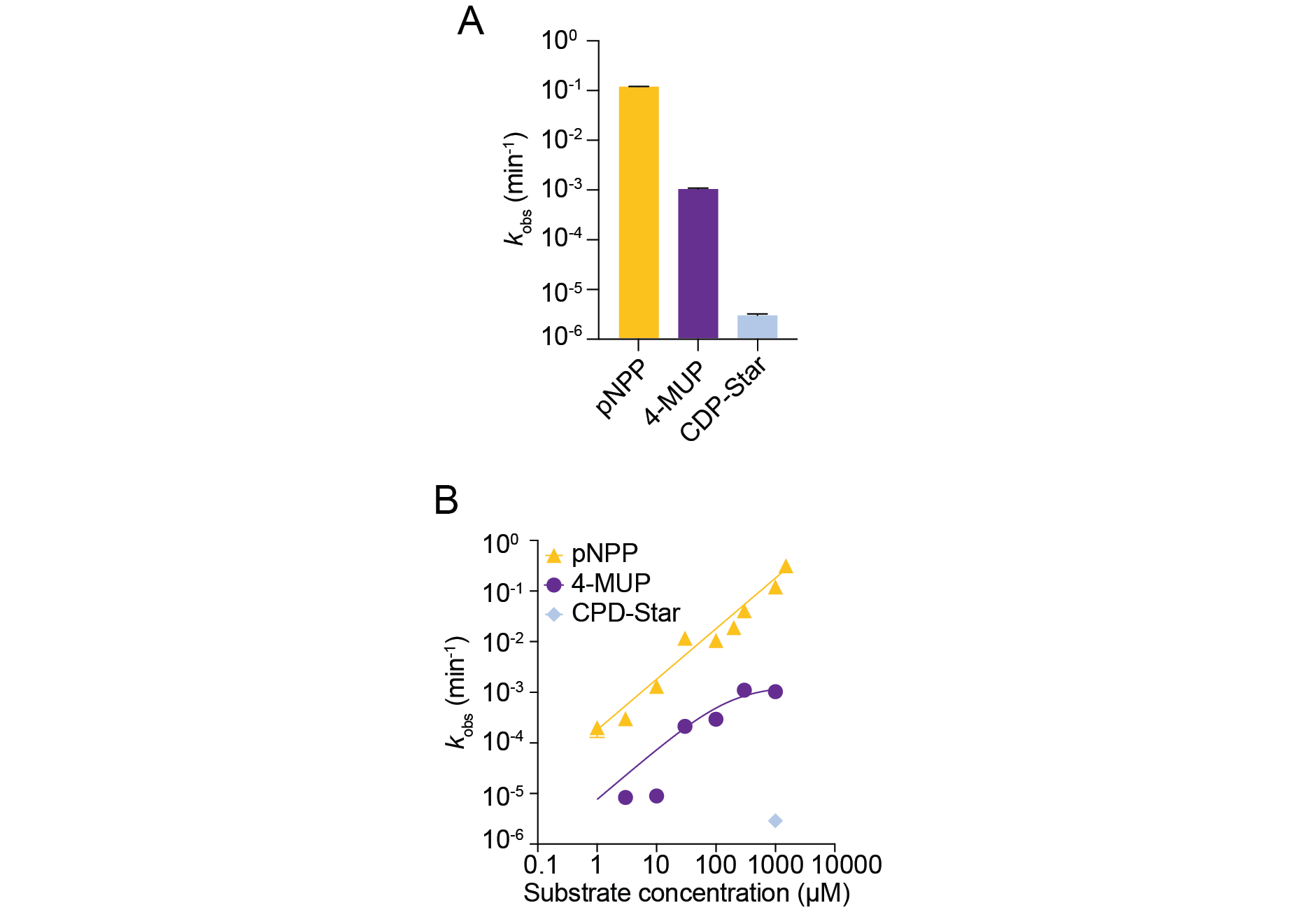


Supplementary Figure 22. Apollon uses 4-NPP more efficiently than 4-MUP or CDP-Star. (A) Catalytic activity of Apollon 2 in the presence of 1 mM pNPP (yellow column), 4-MUP (purple column), and CDP-Star (light blue column). (B) Catalytic activity of Apollon 2 over a range of pNPP, 4-MUP, and CDP-Star concentrations as measured using a ligation assay. Buffers contained 200 mM KCl, 1 mM ZnCl_2_ and 50 mM HEPES pH 7.4. The percent ligated was determined after incubating 1 μM Apollon with the indicated concentration of substrate for 1 hour. Points show average values from three experiments, and error bars represent one standard deviation.


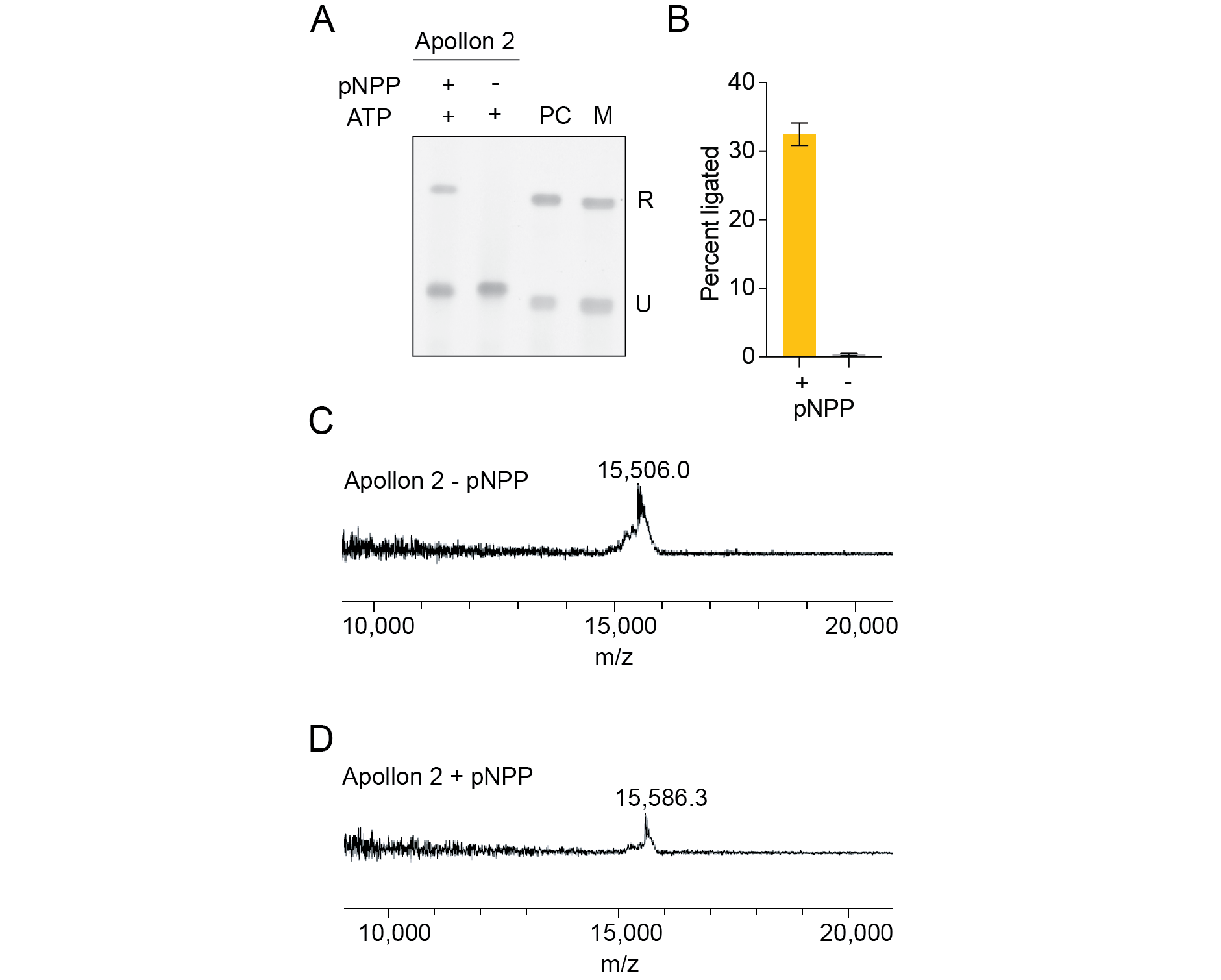
Supplementary Figure 23. Apollon does not react with the ATP in the buffer during the ligation assay. (A) Catalytic activity of Apollon 2 in the presence or absence of pNPP as measured using the ligation assay. After incubating with or without pNPP, samples were ethanol precipitated and then ligated to a short oligonucleotide by T4 ligase in a buffer than contained 1 mM ATP. (B) The percent of ligated Apollon was determined after incubating 1 μM Apollon with or without 1 mM pNPP for 1 hour. Buffers contained 200 mM KCl, 1 mM ZnCl_2_ and 50 mM HEPES pH 7.4. Columns show the average values from three experiments, and error bars represent one standard deviation. Experiments were performed using Apollon 2. (C, D) Same experiment as in panel A, but after incubating Apollon 2 in the presence or absence of pNPP, samples were ethanol precipitated and analyzed by MALDI mass spectrometry.


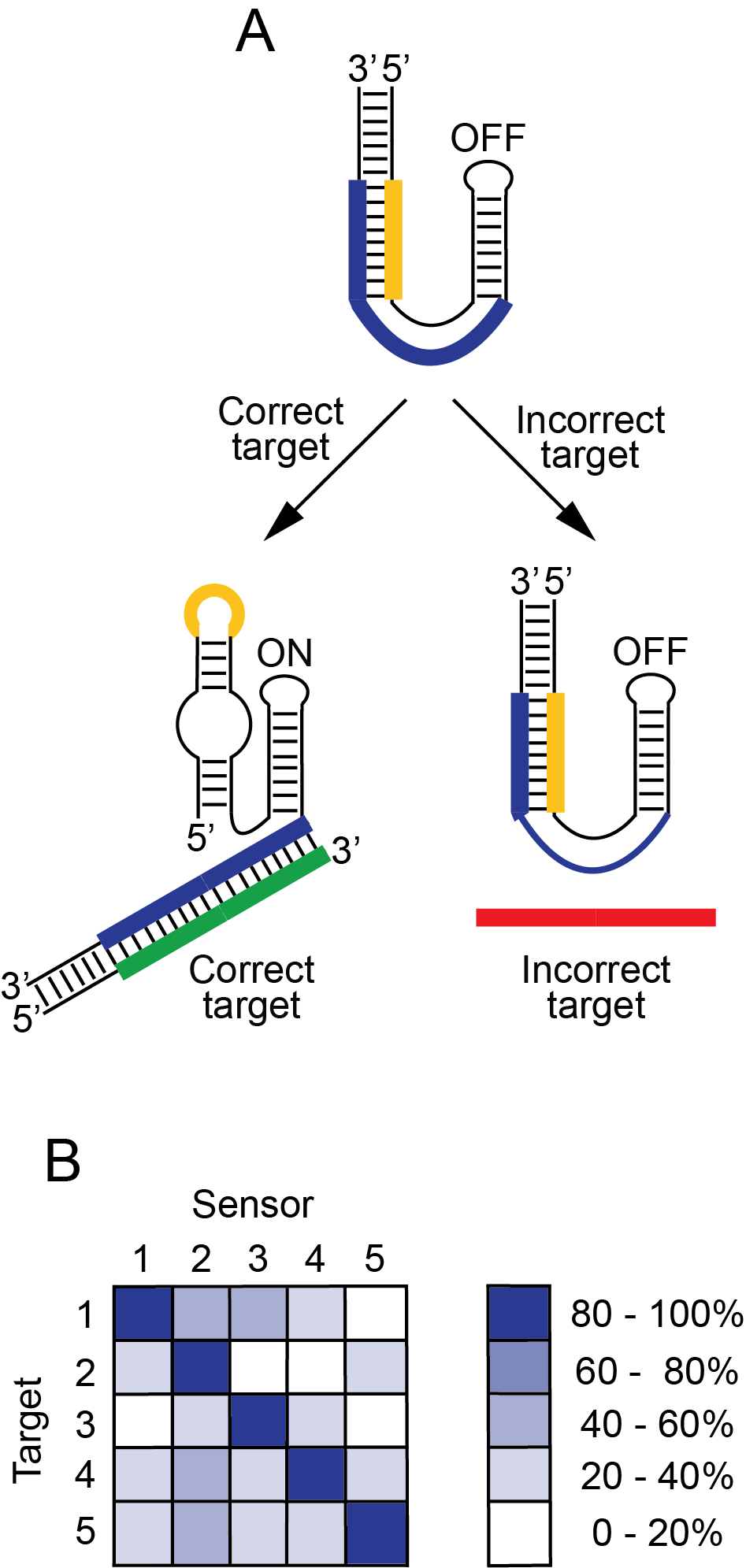
Supplementary Figure 24. Programmable color-producing Apollon sensor that detects oligonucleotides. (A) Detection of oligonucleotides with specific sequences. (B) Experimental characterization of sensor specificity. Five different sensors were generated, each designed to detect a different target oligonucleotide. Each sensor was tested separately in the presence of each of the five target oligonucleotides. These sensors generated more color in the presence of the correct target than in the presence of incorrect targets. Reactions contained 50 μM Apollon oligonucleotide sensor, 100 μM target oligonucleotide, and 100 μM pNPP. The buffer contained 200 mM KCl, 1 mM ZnCl_2_, and 50 mM HEPES pH 7.4. Absorbance at 405 nm was measured after incubating for 24 hours at room temperature using a TECAN Infinite M200 Pro plate reader. The signal to noise ratio is defined as the absorbance at 405 nm in the presence of deoxyribozyme divided by the absorbance at 405 nm in the absence of deoxyribozyme. See Supporting Table 1 for the sequences of Apollon oligonucleotide sensors and target oligonucleotides used in these experiments.


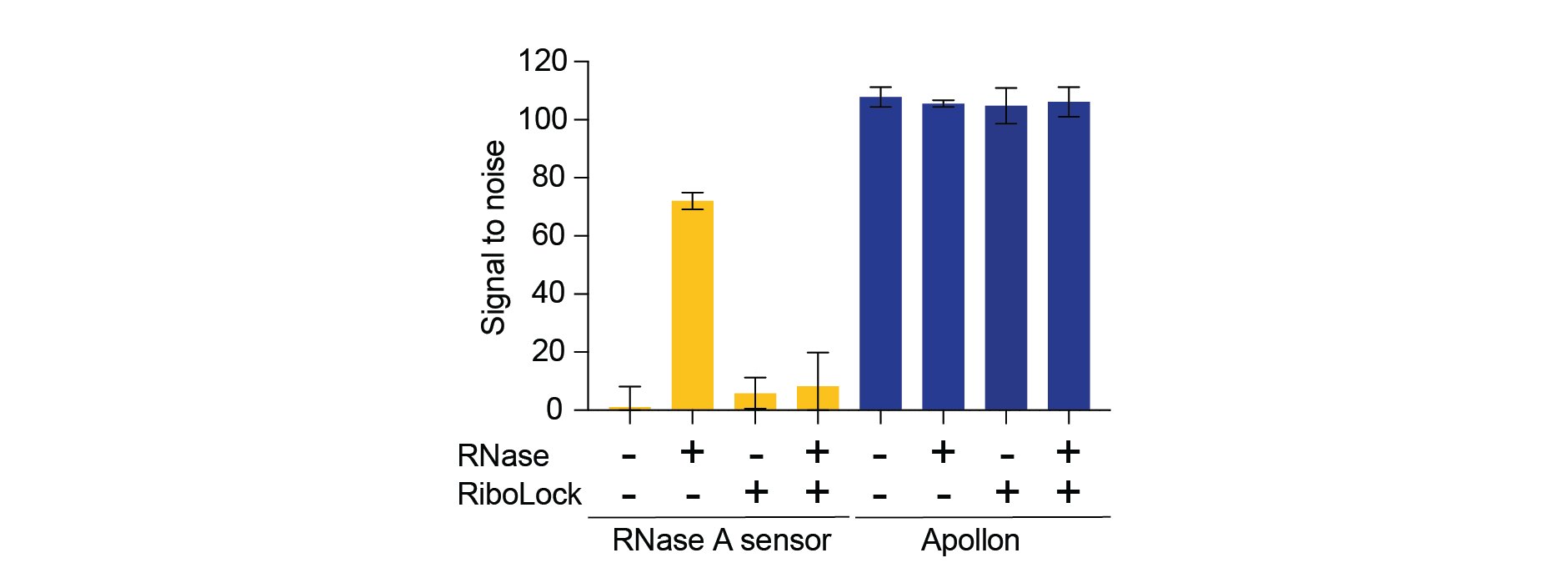
Supplementary Figure 25. A locked Apollon sensor that detects ribonuclease activity. Left: a locked form of Apollon is activated by RNase A, but not when the RNase A inhibitor RiboLock is present. Right: neither RNase A or RNase A inhibitor affects the catalytic activity of unmodified Apollon. Reactions contained 30 μM Apollon ribonuclease sensor, 300 nM RNase A, and 100 μM pNPP. The buffer contained 200 mM KCl, 1 mM ZnCl_2_, and 50 mM HEPES pH 7.4. Color production at absorbance 405 nm was measured for 24 hours at room temperature using a TECAN Infinite M200 Pro plate reader. The signal to noise ratio is defined as the produced absorbance at 405 nm in the presence of deoxyribozyme divided by the produced absorbance at 405 nm in the absence of deoxyribozyme. See Supporting Table 1 for the sequences of deoxyribozymes used in the experiments described in this figure.


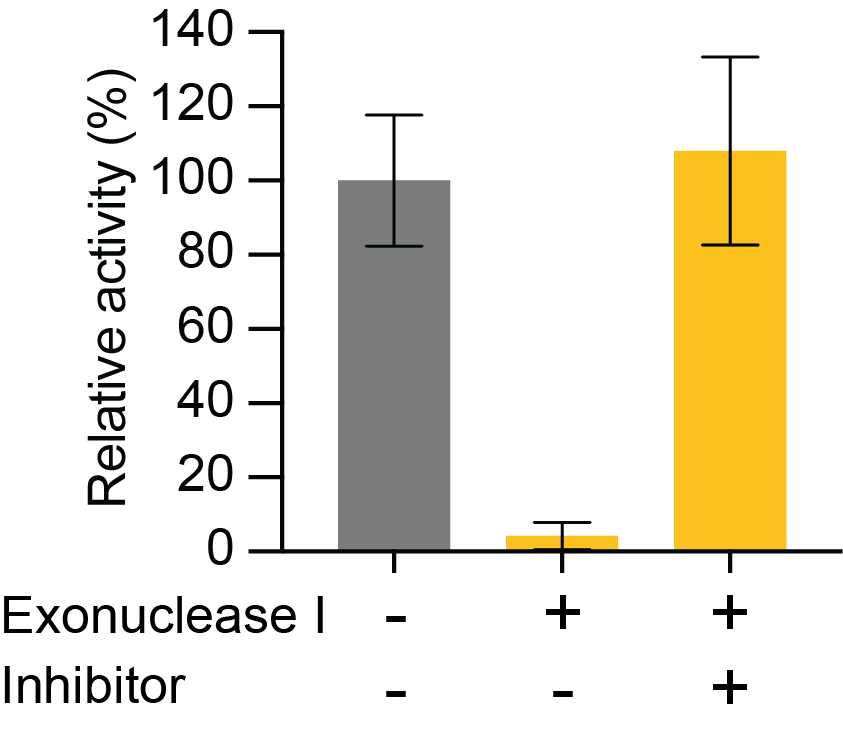
Supplementary Figure 26. Detection of Exonuclease I using Apollon. Apollon is not active after incubating with Exonuclease I, but activity is retained when an Exonuclease I inhibitor is present. 150 μM Apollon 2 was incubated with 60 U of Exonuclease I and 1× Exonuclease buffer (67 mM glycine-KOH pH 9.5, 6.7 mM MgCl_2_, and 1 mM DTT) in a volume of 20 μl at 37°C for 30 minutes to allow Exonuclease I to cleave and inactivate Apollon 2. Following this incubation, 80 μl of 1× Apollon reaction mixture (200 mM KCl, 50 mM HEPES pH 7.4, 1.25 mM ZnCl_2_, and 125 μM pNPP) was added to the reaction. Samples were then transferred to the wells of a clear half-area 96-well plate (Corning). The reaction mixture was incubated at room temperature for 4 hours, and the absorbance at 405 nm was then measured using a TECAN Infinite M200 Pro plate reader. Activity of samples were calculated relative to the activity of Apollon 2 in the absence of Exonuclease I or inhibitor. Columns show average values from three experiments, and error bars represent one standard deviation. Experiments were performed using Apollon 2.
